# Population Levels Assessment of the Distribution of Disease-Associated Variants With Emphasis on Armenians – A Machine Learning Approach

**DOI:** 10.3389/fgene.2019.00394

**Published:** 2019-04-26

**Authors:** Maria Nikoghosyan, Siras Hakobyan, Anahit Hovhannisyan, Henry Loeffler-Wirth, Hans Binder, Arsen Arakelyan

**Affiliations:** ^1^Institute of Biomedicine and Pharmacy, Russian-Armenian University, Yerevan, Armenia; ^2^Research Group of Bioinformatics, Institute of Molecular Biology NAS RA, Yerevan, Armenia; ^3^Laboratory of Ethnogenomics, Institute of Molecular Biology NAS RA, Yerevan, Armenia; ^4^Interdisciplinary Centre for Bioinformatics, University of Leipzig, Leipzig, Germany

**Keywords:** complex diseases, genetic risk alleles, small populations, genome-wide association study, machine learning, self-organizing maps, population-level disease variant distribution, single nucleotide polymorphisms

## Abstract

**Background:** During the last decades a number of genome-wide association studies (GWASs) has identified numerous single nucleotide polymorphisms (SNPs) associated with different complex diseases. However, associations reported in one population are often conflicting and did not replicate when studied in other populations. One of the reasons could be that most GWAS employ a case-control design in one or a limited number of populations, but little attention was paid to the global distribution of disease-associated alleles across different populations. Moreover, the majority of GWAS have been performed on selected European, African, and Chinese populations and the considerable number of populations remains understudied.

**Aim:** We have investigated the global distribution of so far discovered disease-associated SNPs across worldwide populations of different ancestry and geographical regions with a special focus on the understudied population of Armenians.

**Data and Methods:** We have used genotyping data from the Human Genome Diversity Project and of Armenian population and combined them with disease-associated SNP data taken from public repositories leading to a final dataset of 44,234 markers. Their frequency distribution across 1039 individuals from 53 populations was analyzed using self-organizing maps (SOM) machine learning. Our SOM portrayal approach reduces data dimensionality, clusters SNPs with similar frequency profiles and provides two-dimensional data images which enable visual evaluation of disease-associated SNPs landscapes among human populations.

**Results:** We find that populations from Africa, Oceania, and America show specific patterns of minor allele frequencies of disease-associated SNPs, while populations from Europe, Middle East, Central South Asia, and Armenia mostly share similar patterns. Importantly, different sets of SNPs associated with common polygenic diseases, such as cancer, diabetes, neurodegeneration in populations from different geographic regions. Armenians are characterized by a set of SNPs that are distinct from other populations from the neighboring geographical regions.

**Conclusion:** Genetic associations of diseases considerably vary across populations which necessitates health-related genotyping efforts especially for so far understudied populations. SOM portrayal represents novel promising methods in population genetic research with special strength in visualization-based comparison of SNP data.

## Introduction

Non-communicable polygenic diseases such as cancers, neurodegeneration, cardiovascular, and metabolic disorders have become the most prevalent type worldwide and account for the majority of death events in developed and transition economy countries (Habib and Saha, 2010; Benziger et al., 2016). Initiation and development of complex diseases is governed by both, genetic and environmental factors (Ramos and Olden, 2008). Genetic predisposition to complex diseases is not a result of a single mutation, but they require synergic effect of variations in many genes. These variants can be more frequent and/or rare in a population giving rise to “common variant” and “rare variant” hypotheses (Pritchard, 2001; Reich and Lander, 2001). Currently, one of the primary tasks of genome medicine is to identify panels of complex disease-predisposing genetic markers for use in disease prognostics, diagnostics as well as drug development (Abraham and Inouye, 2015).

The most applied method for searching multiple genetic variants is a genome-wide association study (GWAS). During last decades, thousands GWAS have identified numerous single nucleotide polymorphisms (SNPs) associated with different complex diseases such as cancers, schizophrenia and diabetes, Alzheimer’s and Parkinson’s diseases (Giri et al., 2016; Foley et al., 2017; Sud et al., 2017; Billingsley et al., 2018). However, associations reported in one population often do not replicate when studied in another population and, moreover, sometimes they are being reported as neutral or even protective ones (Colhoun et al., 2003; Rice et al., 2007; Li and Meyre, 2013).

The explanation for this fact is that most GWAS employ a case-control design in selected populations, mainly of European, and in lesser extent from African and Chinese origin while other populations largely remain understudied. This issue has gained significant attention during recent years and number of papers has been published which evaluate how risk allele frequencies at known disease loci vary across populations and how this causes biases in population risk score estimation (Jankovic et al., 2010; Abraham and Inouye, 2015; Kim et al., 2018). Moreover, it has been lately shown that assessment of population-level distribution of disease risk alleles can contribute to public healthcare planning (Lau et al., 2018). However, most of this kind of studies either focus on limited population diversity or on a limited set of disease-SNP associations.

Moreover, the inclusion of genetically isolated populations will considerably enhance the understanding of complex trait-associated variants because of their reduced allele diversity (Kristiansson et al., 2008).

In order to describe the entire landscape of population-level variation of diseases-associated SNPs across multiple populations and geographic regions, we used a bioinformatics pipeline based on self-organizing maps (SOM) machine learning. This method has been previously applied to different high-dimensional omics data such as transcriptomic, epigenomic, and proteomic data (Binder et al., 2014; Hopp et al., 2015, 2018; Arakelyan et al., 2017). Its strong visualization capabilities and options for downstream bioinformatics analysis motivated us to apply SOM machine learning to genomic SNP data to study disease-associated risk profiles. We have investigated the distribution of about 44,000 disease-associated SNPs across 52 populations of different ancestry and geographical origin; among them the so-far understudied population of Armenians. Historically inhabiting the region of the South Caucasus, Armenian population was reproductively isolated since the Bronze Age (Haber et al., 2016), which makes them an interesting example for studying local specifics of the interaction of distribution of genetic risks for complex diseases with actual disease prevalence on the population level.

## Materials and Methods

### Data and Pre-processing

**FIGURE 1:**
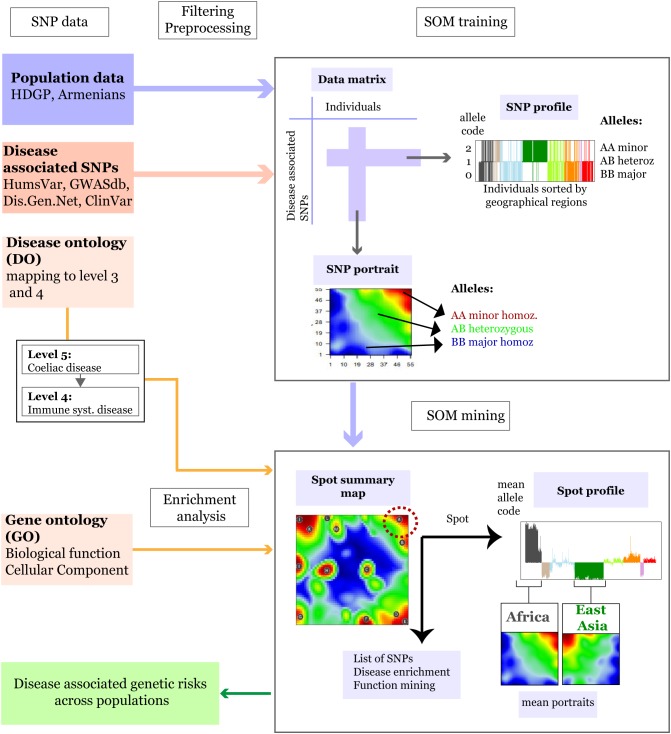
Schematic overview of the SOM-portrayal method applied for analysis of population SNP data (see section “Materials and Methods”). SNP data of different populations around the world taken from the Human Genome Diversity Project (HDGP) and of a cohort of 99 Armenians were filtered for disease associated SNPs collected from four databases (see section “Materials and Methods”). Then, the data matrix (44,234 SNPs × 1039 individuals) was used to train a SOM which delivers a SNP portrait of each individual. It represents a colored image showing clusters of SNPs with increased minor allele frequency (MAF) as red ‘spot-like’ areas. They were then used for extracting population-specific associations with disease risks and biological functions by applying enrichment techniques. The SOM mining step also makes use of overview maps summarizing all spots observed on population averaged mean portraits which characterize the SNP landscape of individuals of a certain geographic region and of SNP profiles showing the allele score across all individuals and populations studied. For example, the red minor-allelic spot in the right-upper corner of the map (see dashed circle) is specific for Africans because it is observed in their portraits but not in the portrait of Europeans. Its profile shows high and low values of the allele score for individuals from these regions. Each of the spots delivers a list of SNPs and associated genes, which, in turn, are used to extract disease risks for populations showing these spots.

In the first step of analysis population-related SNP data were merged with disease-associated SNPs and preprocessed (Figure 1). We considered the following data sets.

#### Population Data (HGDP and Armenians Data Set)

We used preprocessed genome-wide SNP data (Illumina 650Y arrays) taken from the Human Genome Diversity Project (HGDP^1^) after removal of atypical and duplicated samples. The data collect genotypes (650,0000 SNPs) from 940 individuals from 51 populations in 8 geographical regions (Africa, Europe, Middle East, South and Central Asia, East Asia, Oceania, and America) (Rosenberg, 2006).

Single nucleotide polymorphisms data (Illumina Human Omniexpress microarray platform) of 99 Armenians (Eastern Armenian population) was taken from the recent publication by Haber et al. (2016).

#### Disease-Associated SNP Data

Lists of SNPs associated with diseases were collected from the following four databases: UniProt humsavar^2^, NCBI Clinvar^3^, GWASdb^4^, and DisGenNet^5^. The lists from all sources were then combined and the duplicated records were removed. The final list consisted of 321,955 disease-associated SNPs.

#### SNP-Filtered Population Data

Disease-associated SNPs with minor allele frequency (MAF) > 0.05 were selected from both data sets after removing missing genotypes using “vcftools.” VCF genotype files were transformed into genotype matrix using “variant annotation” (Obenchain et al., 2014) and “snpstats” (Guino et al., 2006) R packages. Final dataset consisted of 44,234 disease-associated SNPs in 1039 samples that combined HGDP and Armenian populations.

#### Allele Coding

For further data processing, SNP-genotypes were coded by the following integers: 0 – homozygous major alleles genotype, 1 – heterozygous alleles genotype, and 2 – homozygous minor allele genotype. The full set of SNPs of each individual constitutes its SNP-portrait while the allele-coded values of each SNP over all individuals in the data set constitute its SNP-profile (Figure 1).

#### Disease Classification

We used Disease Ontology (DO, release 2018-07-05) based classification of diseases. The DO is structured into types of disease on different levels using a tree-model (Schriml et al., 2012). For comparability of disease-SNP associations, we mapped DO-terms of level 4 and higher to level 3 of DO terms. For instance, diabetes mellitus (level 5) is assigned to carbohydrate metabolism disease (level 3) in further analysis (Supplementary Figure S1).

### Generating SNP-Portraits Using Self-Organizing Maps

In the next step preprocessed and filtered HDGP SNP datasets were feature centralized and then clustered using SOM machine learning (see Wirth et al., 2011 for a detailed description of the method, and Figure 1 for a schematic representation). It translates the original data matrix consisting of the allele scores of *N* = 44,234 disease-associated SNPs collected from *M* = 1,039 individuals into a data matrix of reduced dimensionality of *K* = 3,025 so-called meta-SNP profiles. Hereby, the term ‘profile’ denotes the vector of allele score values across the individuals. The SOM training algorithm distributes the SNPs over the K micro-clusters of meta-SNPs by minimizing the Euclidian distance between the SNP-profiles as a similarity measure. This ensures that SNPs with similar profiles cluster together in the same or in closely located meta-SNPs. Each meta-SNP profile can be interpreted as the mean profile averaged over all SNP profiles referring to the respective meta-SNP cluster. The allele scores of the meta-SNPs of each individual are visualized by arranging them into a two-dimensional *M* = 55 × 55 grid and by using red to blue colors for a maximum to minimum allele score values in each of the grid images. These images ‘portray’ the genetic landscape of each individual studied. We used SOM implemented in “oposSOM” R package (Löffler-Wirth et al., 2015). All populations were labeled according to the geographical location while Armenians were considered as a separate group. Mean SNP-SOM portraits of populations from the same geographic regions were obtained by averaging the meta-SNP values of the respective individual SNP-portraits. A separate “zoom-in” SOM (Wirth et al., 2011) was trained by considering only populations of the HDGP data set from the Middle East and Europe together with Armenians to better resolve details of their disease-associated genomes. Full data analysis results are available from Zenodo Open data platform (Nikoghosyan et al., 2018).

### Spot Clustering, Disease, and GO-Term Enrichment

In the third step, we performed an analysis of the SOM-clustered data to assess disease-associated genetic risks across the populations. Our SOM implementation used a ternary code for coloring each meta-SNP giving rise to spot-like red and blue colored regions in the SNP-portraits due to the self-organizing properties of the SOM algorithm. Red and blue spots refer to minor and major allelic regions while green areas mark heterozygous alleles. We then used segmentation algorithms developed previously (Wirth et al., 2011) to extract so-called spot-clusters of (red) minor-allelic regions. Each of these spot-clusters consists of 100 to 1000s of SNP-profiles. Enrichment of disease DO terms in the spot clusters was then estimated by Fisher’s exact test. For each spot, the test assesses whether the number of SNPs associated with a given disease is larger than expected under the assumption of random distribution of SNPs among the spots. Enrichment analysis was also performed for gene-ontology (GO) terms “biological process” and “cellular component” using over-representation analysis as implemented in WebGestalt web-server (Wang et al., 2017) to assess the functional context of the genes containing the SNPs in a given spot.

## Results

### SOM-Portrayal of Geographical Diversity of Disease-Associated SNPs

**FIGURE 2:**
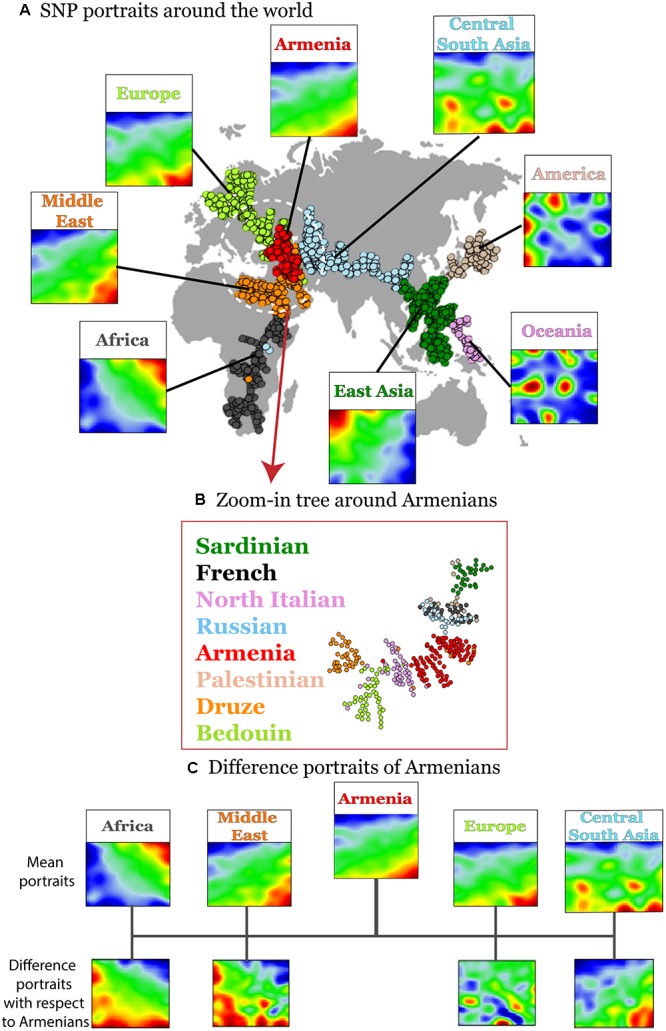
Variation of disease-associated SNP portraits across geographic regions. **(A)** Mean SNP-portraits of seven geographic regions show systematic changes of their spot patterns. A minimum spanning tree (MST) was calculated using Pearson’s correlation coefficient between the SOM portraits of the individuals to visualize the similarity relations between their SNP-patterns. It is mapped on the geographic map to illustrate the relation between the genetic drift and the geographic distribution of the populations. Each circle refers to one individual. Their colors assign the respective geographic region. Armenians (red) form a cluster at the crossroad between African, European, and Asian branches of the MST. **(B)** A zoom-in SOM was calculated using data of selected populations from Middle East, Europe and Armenians for SOM-training to better resolve local similarity relations. The zoom-in MST reveals a relative compact clusters of Armenians bordered by populations from Middle East and Europe, respectively. **(C)** Difference portraits of Armenians with respect to other populations show an increase of non-African genetic contributions with respect to Middle Eastern populations and increased European contributions with respect to Central and South Asian populations.

Human disease-related genetic diversity is shaped by demographic, biological, and environmental factors. Here we applied a SOM approach to gain new insights about population-level distributions of disease-predisposing alleles across geographic regions using whole genome SNP-scans of 1039 individual selected from 52 ethnicities in seven geographic regions and of Armenians considered separately. SOM was trained using ca. 44,000 disease-associated SNPs. We obtained a gallery of “SNP portraits” visualizing the genotypes of disease-associated SNPs for each individual studied (Supplementary Figure S2). Inspection of the portraits reveals high diversity of textures reflecting the allelic landscapes in terms of areas enriched for major homozygous, heterozygous and minor homozygous genotypes color-coded in blue, green, and red, respectively. On the other hand, sample portraits were mostly very similar for individuals originating from the same geographic region while the portraits of individuals from different regions progressively diverge with increasing geographic distance in most cases. For example, individuals from sub-Saharan Africa typically show a red “spot” in the right upper corner of their SOM-portraits which shifts toward the right lower corner for individuals from Middle East and Europe including Armenians. This shift reflects the fact that the latter three populations show on average similar collections of minor homozygous disease-associated SNPs which however differ markedly from those of Sub-Saharan Africans. The red spots in the mean portrait of individuals from Central and South Asia partly overlap with those of Europeans but it shows also new, ubiquitous spots referring to disease-risk associated SNPs not observed in Europeans. Also the mean portraits of East Asian, Native American, and Oceania populations reflect a combination of common and ubiquitous spots reflecting footprints of their population history. To visualize the similarity relations between the individuals from different geographic regions we generated a minimum spanning tree (MST) based on Pearson’s correlation coefficients between their SOM-portraits (Figure 2A). For comparison we generated an independent component analysis (ICA) plot which is often applied as similarity presentation in population genetics (Supplementary Figure S3). The results reflect the variation of disease-associated alleles across the geographic regions. Interestingly the MST resembles also the distribution of the populations across the geographic regions ranging from Africa at one end to America and Oceania at the other one. A similar MST was recently reported by us using a selection of most variant SNPs instead for disease-associated ones (Binder and Wirth, 2014). The disease-associated SNP-genotypes selected here reflect similar genetic drift effects as the most-variant SNPs.

Interestingly, the Armenian individuals accumulate into a homogenous cloud at the crossroad between three branches collecting populations from (sub-Saharan) Africa and Middle-East, from Europe and from Asia, respectively. This localization of the Armenian cluster is in accordance with the previous genetic studies based on the genetic variation data on autosomal and uniparental loci (Hovhannisyan et al., 2014; Haber et al., 2016; Yepiskoposyan et al., 2016).

A more detailed view using a zoom-in SOM using only populations from the Middle East and Europe further emphasize the intermediate position of the Armenian population in-between the Middle East and Europe (Figure 2B). Difference portraits show that disease-associated allele-landscapes of Armenians are characterized by non-African patterns compared with Middle East populations and by European patterns compared with Central and South Asian populations. The difference in comparison to other European populations is subtler showing also marked similarities in the allelic composition. In summary, SOM-portrayal of disease-associated SNPs reflects and characterizes the distribution of humans across geographical regions. Armenians occupy a central position of their disease-associated genome between Middle Eastern, European, and Central Asian populations in a region of an ancient crossroad of human migration.

### Segmenting the SNP Landscape Into Minor-Allelic Spots

**FIGURE 3:**
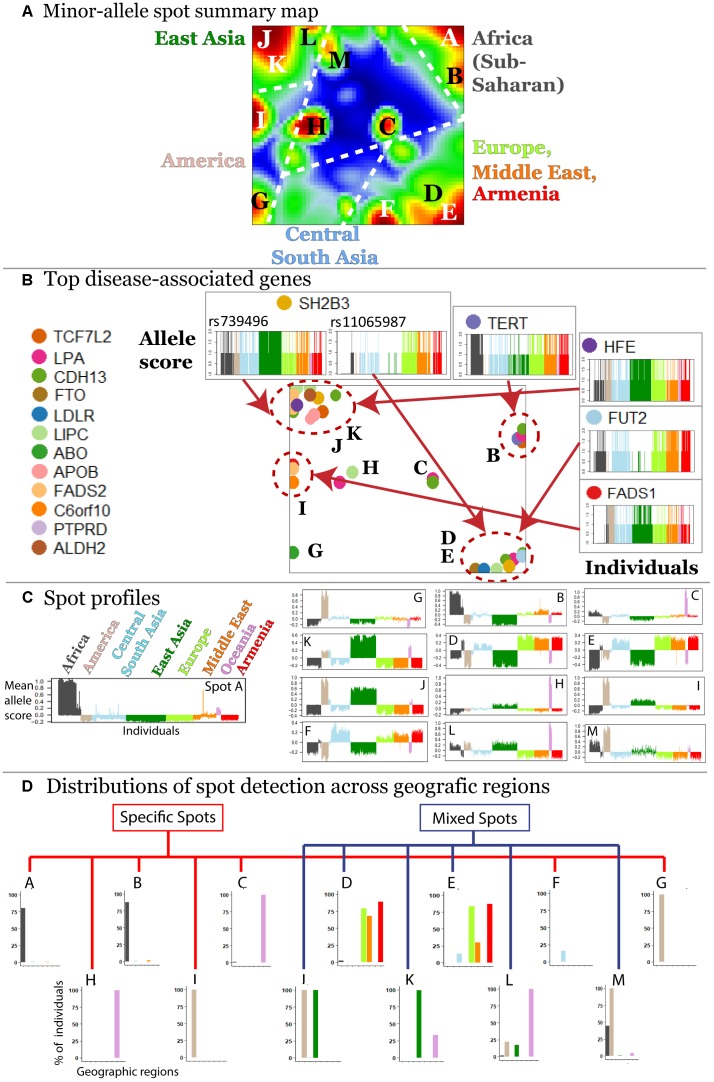
Characterization of the SNP landscape. **(A)** The spot summary map segments into 13 spots (see labels A–M) enriching minor-alleles in a population/geography specific way. **(B)** The positions of 17 SNPs referring to different genes selected from the top 40 genes having a high number of disease associations (Price et al., 2015) in the SOM are indicated by arrows. Accumulation of these genes in the spot areas is indicated by dashed circles. The profiles of selected SNPs reveal a population-specific enrichment of minor-allelic scores. **(C)** Mean allele profiles of the SNPs collected in each of the spots are shown as barplots. Each of the spots refers to a different profile. **(D)** Part of the spots can be assigned to one geographic region while ‘mixed’ spots show enrichment of minor alleles in populations from more than one region (see also the mean portraits in Figure 2A).

The majority (about 70%) of minor alleles in the HGDP dataset associated with the diseases studied, which is in accordance with previous observations (Lachance, 2010). We were interested to study clusters of co-localized minor-allelic SNPs that are evident as red, spot-like areas in the SNP-portraits. The spot summary map collects all relevant red spots (clusters of SNPs with minor allele high frequency) to provide an overview of the minor-allelic spot regions observed in the mean SOM portraits of the different geographic regions (Figure 3A). Overall we identified 13 minor-allelic spots labeled by capital letters A–M (Figure 3A). Mean profiles averaged over the allelic codes of all alleles collected in the respective spot reveal the geographic specificity of minor allele prevalence (Figure 3C). We identified seven spots which were unique for a given geographic region and another six (mixed) spots which shared between several regions (Figure 3D). For instance, portraits for Armenians, Europeans, Central South Asians and populations from the Middle East are characterized by red spots located in the right-lower corner, while the portraits from (sub-Saharan) Africa and from East Asia show different spots in the right and left upper corners of the map, respectively. On the other hand, SNP portraits from Oceania and, to a lesser degree, from America are characterized by two or more spot both unique and/or mixed distribution. For example, spot L reflects similar minor allelic SNP profiles of Oceanians, Native Americans and East Asians and partly also Africans while spot I reflects common genetic history of original populations in America and East Asia.

In order to demonstrate how SOM assigns single SNPs into clusters based on their allele frequency profiles, we mapped 40 SNPs from 17 genes with a high number of disease associations taken from Price et al. (2015) into the SNP landscape (Figure 3B). The most of the SNPs accumulate in the regions of spots D and E (19.5%) and of spots J and K (34.1%) corresponding to European and East Asian populations, respectively. About 38% of the genes were found in or near spots assigned to minor allele enrichment in other geographic regions such as Africa, Oceania, and America. This unbalanced distribution is presumably due to the population bias in the studied SNPs toward Europe and East Asia. It emphasized the necessity of extending genetic association studies to other populations.

We also evaluated the effect of linkage disequilibrium (LD) on distribution of SNPs in the SOM portraits, using SNPs located on chromosome 1 available in our dataset. SOM algorithm naturally tries to allocate SNPs with correlated profiles in close proximity (or in the same cluster) while SNPs with anti-correlated profiles are positioned in furthest regions of the SNP portrait. Thus SNPs that are in LD will be either located in one cluster (for positively associated alleles) or in two clusters located most distantly on the “SNP portrait” (for negatively associated alleles) (Supplementary Figure S4). Furthermore, since the disease-associated SNPs used in our study were already “pre-selected” based on GWAS or functional studies, and since the goal of our study was “portraying the population-level genetic risks” for known associations rather than identifying new ones, we can assume that LD’s effect on our findings was minimal.

Thus, the SOM method aggregates disease-associated alleles into clusters associated with one or more regions this way reflecting geographical variability of disease susceptibility coded in MAF.

### Associations Between Diseases and Spot-Modules of SNPs and Their Functional Context

Next, we evaluated disease enrichment in spots compared with their background distribution based on the clustered SNPs using Fisher’s exact test. This “background” distribution shows that the largest number of SNPs is associated with complex diseases such as cardiovascular, nervous and respiratory system disorders and carbohydrate metabolism disease (Supplementary Figures S5, S6).

**FIGURE 4:**
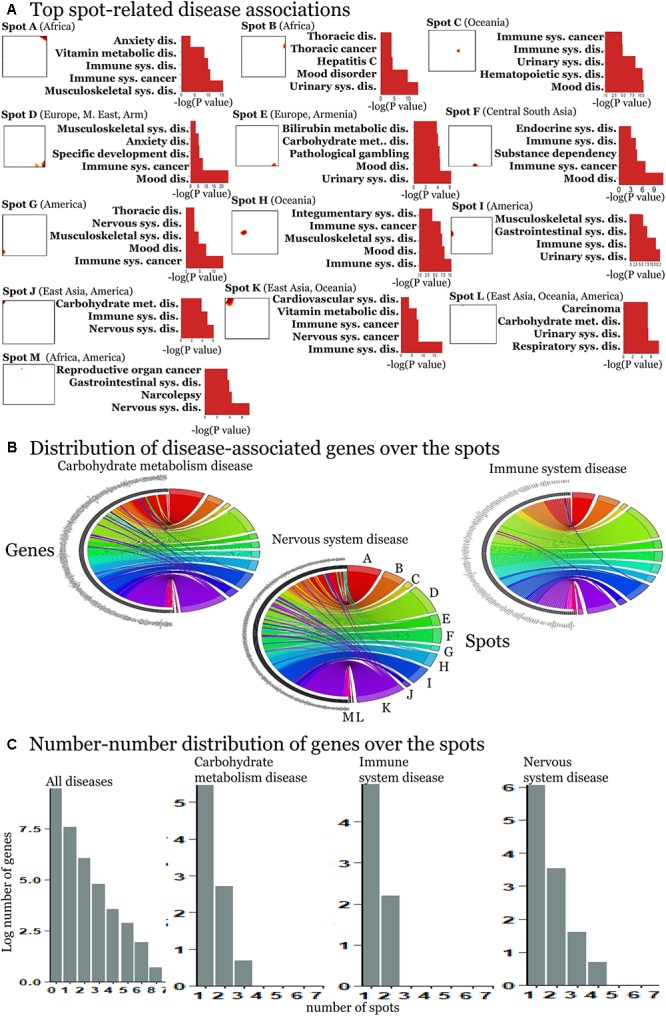
Spot-enrichment of disease-associated SNPs. **(A)** Top enriched disease terms in each of the spots (see Supplementary Figures S7–S19 for full lists of enriched diseases and Supplementary Figures S5, S6 for their background distribution). Enrichment *p*-values are obtained using Fishers exact test. **(B)** The circular plots link genes with spot-clusters containing SNPs referring to these genes. Each circular plot shows SNPs which associate with one disease. Different genes which associate with the same disease distribute over different spots. Genes specifically accumulate in the spots in a one-to-one fashion as a rule of thumb. Only a few genes were found in two or more spots according to different SNPs in the same gene (e.g., SH2B3). Examples were shown for three selected disease classes. **(C)** The number–number distributions over the spots follow an exponential decay meaning that the majority of genes associates with a single spot.

We detected 11 significant disease associations per spot on the average (Supplementary Figures S7–S19). The top diseases per spot are presented in Figure 4A. Hereby the same diseases such as carbohydrate metabolism disease (diabetes mellitus), mood disorders, immune system, and neuronal diseases enrich in different spots. These redundantly distributed diseases typically associated with SNPs in different genes as shown in the plots in Figure 4C. They revealed predominantly a one-to-one relation between the SNPs in spots and diseases (Figure 4B). The distribution of genes counts over the spots (Figure 4C) roughly follows an exponential decay law meaning that the number of genes associated with one spot dominates over the number of genes associated with multiple spots.

**FIGURE 5:**
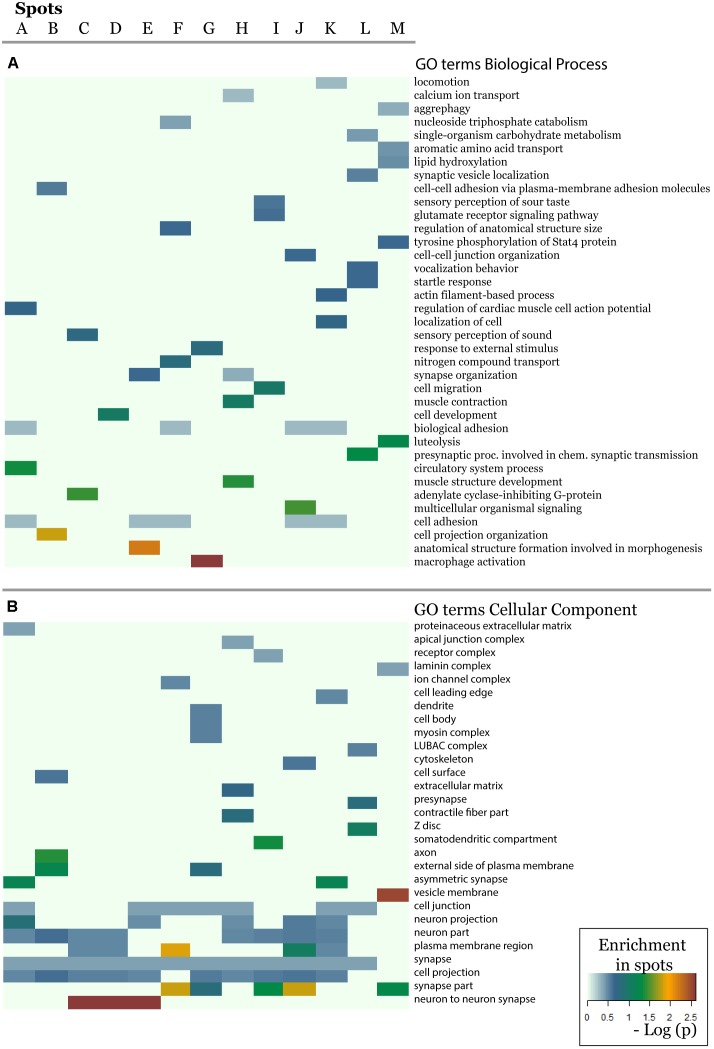
The biological context of the spots is estimated by means of enrichment analysis using the GO terms **(A)** biological process and **(B)** cellular component. Enrichment *p*-values (Fisher’s exact test) were visualized as heatmap. One sees that, e.g., GO-terms related to neuronal function are enriched in most of spots but especially in spots showing minor allele preference in Europeans.

In order to further assess the difference on the functional context of the genes carrying the SNPs, we performed functional annotation of GO in each spot using over-representation analysis implemented in Webgestalt web-server (Wang et al., 2017). The results demonstrate that each spot is characterized by an almost unique set of enriched GO biological process terms (Figure 5A). Similar patterns are observed in the enrichment of GO terms related to molecular function (Supplementary Figure S20) and cellular localization (Figure 5B). On the other hand, one finds the same terms [e.g., related to adhesion, which plays an important role in maintaining the physiological state of various organs (Müller, 2006)] in different spots. Other GO-terms enriched in the spots included cell migration, cell and organ development and signaling which all appear to be deregulated in various diseases.

Our results underpin the complex character of diseases pathophysiology, which involves deregulations in multiple biological pathways and cellular networks (Zheng et al., 2018) often in a population-specific fashion (Ran et al., 2011). In summary, our results demonstrated considerable specificity of the distribution of genes and biological processes associated with the same diseases at the geographic levels.

### Genetic-Risk Profiling

**FIGURE 6:**
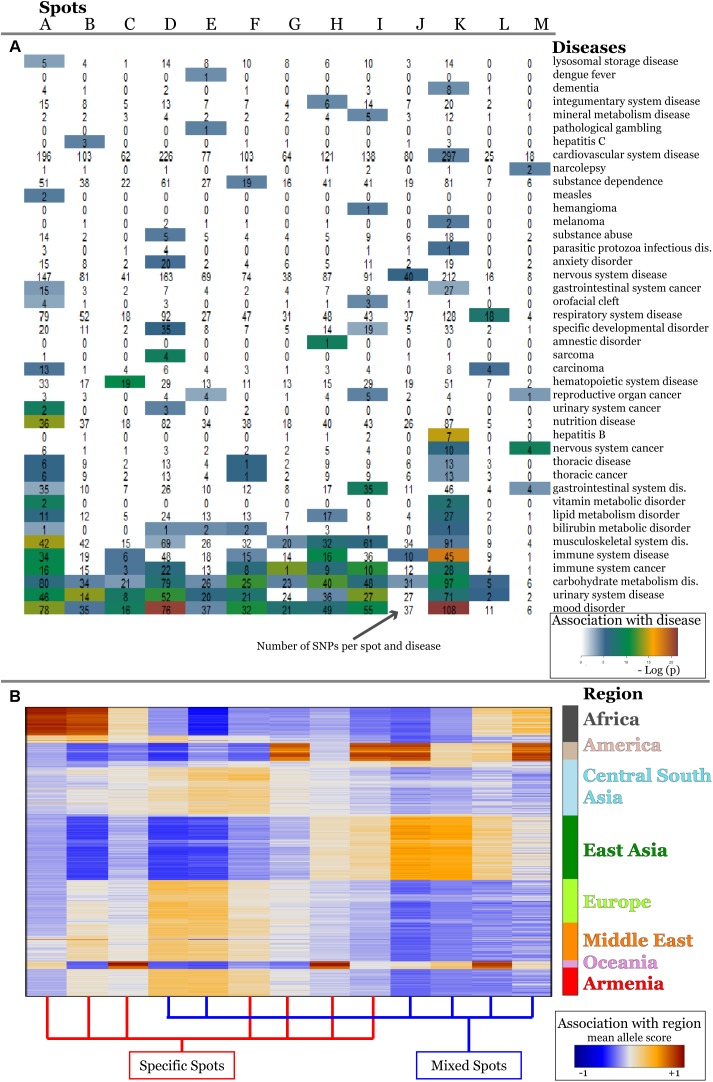
Associations of spots with diseases **(A)** and with geographic regions **(B)** are shown as heatmaps. Diseases represented with high numbers of SNPs in the background distribution accumulate in the lower part of the heatmap **(A)**. More rarely-presented diseases frequently associate with one or two spots only **(A)** which, in turn, are assigned to specific regions according to the respective mean allele score **(B)**. SNPs associated with mood disorders are enriched in spots having high MAF in Europeans and East Asians which corresponds to the enrichment of GO-terms related to neuronal functions in Figure 5.

For the detailed overview, we represented the disease-spot associations as a heatmap in Figure 6A. We compared them with the minor allele score profiles of the spots (Figure 6B) to combine the assignment of diseases with geographic regions.

The diseases accumulating in the lower part of the heatmap in Figure 5A are the most thoroughly studied ones showing highest overall enrichment in the background distribution (compare with Supplementary Figure S5) as well as in spots. According to the minor allele enrichment, these diseases can be considered as the most prevalent ones worldwide. Indeed, the global prevalence of diabetes (carbohydrate metabolism disease) is 8.5% (Kakkar, 2016), which makes it one of the most frequent diseases. Mood disorders (bipolar disorder, anxiety, and depression) are considered as the most frequent mental conditions (Steel et al., 2014), while immune system cancers (mostly malignant diseases of blood and lymphoid system) also have been reported to have high incidence rate worldwide (Foreman et al., 2018). Thoracic cancer (including lung cancer) associated SNPs were significantly enriched in three spots (A, E, K) covering all geographic regions.

The diseases in the upper part of the heatmap in Figure 6A are less enriched in the background distribution and thus they refer to moderately prevalent/studied ones. These diseases reveal region specificity of spot enrichment.

For example, vitamin metabolic disorders associated SNPs were enriched in spots A and K showing increased minor allele scores in Africa and East Asia, respectively. Vitamin deficiency in these regions was mostly attributed to economic and political reasons and also to local dietary practices (Bailey et al., 2015). Our results, however, showed that four SNPs (rs1348864, rs4778359, rs7781309, rs9937918) which associate with vitamin D metabolism (Bernatzky et al., 2009; Engelman et al., 2010) show high MAF in Africa and East Asia suggesting also increased genetic risk. Notably, for these regions, low levels of the vitamin D deficiency marker 25-hydroxyvitamin D in blood were reported (Prentice, 2008; Prentice et al., 2009).

Likewise, SNPs for bilirubin metabolic disorder accumulate in five spots (A, D, E, F, and K) linked to Africa, Europe, the Middle East, Asia, and Armenia. Interestingly, previous studies clearly implicated SNPs identified in the spots with the serum bilirubin levels in Europeans, Asians, and Africans (Kim et al., 2010; Chen et al., 2012; Cox et al., 2013). Moreover, population-dependent sets of mutations and polymorphisms were shown to be implicated in the development of inherited disorders of bilirubin clearance (Memon et al., 2016).

Finally, we found that some diseases are enriched in a single spot. For example, SNPs related to anxiety disorders were significantly enriched in Europe, the Middle East and Armenia (spot D). This result is in line with the large-scale meta-analysis performed by Baxter et al. (2013) indicating significantly reduced risk for anxiety disorders in non-western cultures compared with the western ones.

Overall, the results of population levels genetic risk profiling indicate a bias toward more prevalent diseases with global impact, such as cancers, immune system diseases, and diabetes. This, in turn, results in a larger number of associations, compared with less widespread diseases. We also find that the enrichment of diseases associated SNPs links to the disease prevalence in many cases.

### Genetic Risks of Armenians

The analysis of the global SNP-landscape of worldwide populations provided an overview of the geographic distribution of disease-related genetic risk factors. However, it virtually does not resolve finer-granular population-level diseases-associations, especially, for relatively small populations such as Armenians. Our initial analyses reveal patterns of disease-associated alleles that they share with neighboring populations from the Middle East and Europe (e.g., Spots D and E in Figure 3A).

A detailed comparison of SNP portraits showed that Armenians are characterized by different spot patterns compared with that observed for populations from Europe, the Middle East and Central South Asia (Figure 2C).

In order to better resolve differences between these neighboring populations, we performed a so-called zoom-in SOM-analysis (Wirth et al., 2011) that considered only populations from Europe (French, Sardinian, Russian, North Italians), Middle East (Bedouin, Druze, Palestinians) and Armenia.

**FIGURE 7:**
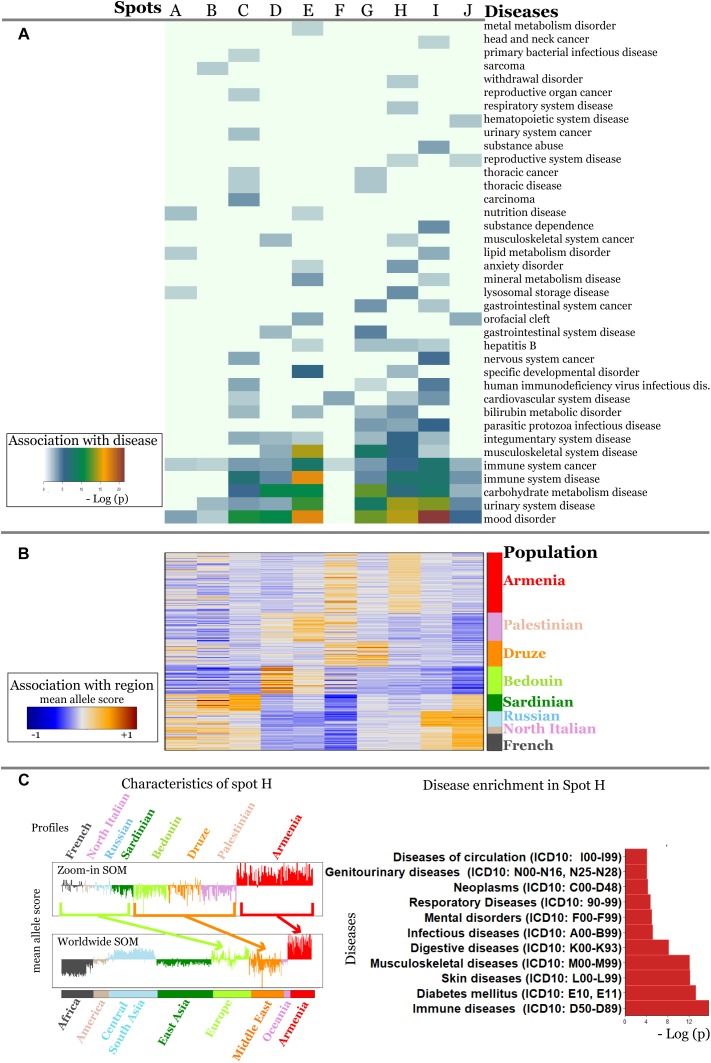
Zoom-in analysis of selected populations from Europe and Middle East and of Armenians resolve geographic specifics of association between SNPs and disease risks with higher resolution. **(A)** Spot enrichment of diseases and **(B)** of minor allele scores are shown as heatmaps. Notably, spot H collects SNPs which suggest specifically increased disease risks in Armenians. **(C)** Profiles of spot H in the zoom-in and the world-wide SOM indicate specifically increased MAF of the included SNPs for Armenians. Top diseases which associate with spot H are shown as barplot of enrichment *p*-values.

It revealed a spot cluster of minor alleles of SNPs which specifically characterize Armenians (spot H in Figure 7). These SNPs associated with immune diseases, diabetes mellitus, skin diseases, and musculoskeletal diseases as the top-four ones. The top SNPs showing highest MAF, the affected genes, associated diseases and their incidence in Armenia (Andreasyan et al., 2017) are listed in Table 1.

**Table 1:** Top disease-risk associated SNPs in Armenians.

SNP-ID^∗^	Genomic region/GENE SYMBOL	REF/ALT	MAF Armenia	MAF other	P ASSOC	DO_TERM	Prevalence per 100000 in Armenia
rs2848713	Intergenic	G/A	0.21	0.1	5.6E-45	Behcet’s disease	NA
rs2298075	SEC31B	C/A	0.35	0.2	2E-04	Breast cancer	66
rs10166672	Intergenic	A/G	0.24	0.15	9E-04	Alzheimer’s disease	NA
rs627834	Intergenic	A/G	0.25	0.07	3.3E-04	Alzheimer’s disease	NA
rs10952163	Intergenic	A/G	0.22	0.08	8.6E-05	Obesity	NA
rs17213431	ANLN	G/A	0.26	0.1	4.4E-05	Obesity	NA
rs11669309	LOC284395	C/T	0.21	0.09	9E-04	Hypertension	5643
rs4239131	Intergenic	T/C	0.2	0.09	4.4E-04	Gilles de la Tourette syndrome	NA
rs4239131	Intergenic	T/C	0.2	0.09	4.4E-04	Obsessive-compulsive disorder	NA
rs4239131	Intergenic	T/C	0.2	0.09	4.4E-04	Attention deficit hyperactivity disorder	NA
rs7022505	Intergenic	C/T	0.22	0.12	3.7E-06	Kidney disease	NA
rs10237038	FLJ43663	C/T	0.22	0.11	2.7E-05	Pancreatic cancer	32
rs2269706	PPP1R18	G/A	0.21	0.16	6.3E-06	Rheumatoid arthritis	865


*^∗^SNPs were located in spot H (Figure 6) and further sorted according maximum ratio MAF (Armenians)/MAF (others)*.

For example, the incidence of Behcet’s disease was reported to be higher in Armenians and other South Caucasus populations compared with Russians (Lennikov et al., 2015). The highest prevalence of this disease has been reported among Turkish (450–500 per 100000), however, the prevalence of 90 per 100000 in ethnic Armenians (Oke and Khulief, 2016) is still considerably higher compared to Europeans (Leonardo and McNeil, 2015).

Toutette syndrome SNP is among the disease SNPs associated with Armenians. Systematic studies considering 44 populations have reported that Tourette syndrome is rare among Afro-Americans in the United States and sub-Saharan Africans. Till date, most of the Tourette syndrome cohorts have been described from Western sites and also from China, Japan, and the United Arab Emirates (Qi et al., 2017).

Currently no data is also available about the incidence and prevalence rates of Alzheimer’s disease in Armenia, however, it is accepted that the actual rates are comparable if not higher compared with worldwide rates (Saberi et al., 2012; Tataryan, 2012). Official statistics is also unavailable for obesity; however, the 2013 report by WHO (WHO, 2013) indicated that 55.5% of the adult population in Armenia were overweight and 24.0% were obese. Overall our analysis suggests links of population-level enrichment of diseases associated alleles and disease prevalence particularly in Armenians, which however presently lack reliable data about disease prevalence.

## Discussion

In this study, we analyzed population and geographic region-wide distributions of disease-associated genetic risk factors using SOM machine learning. This approach generated region and population specific “SNP portraits” visualizing the distribution of disease-predisposing alleles and allowed for direct comparisons and assessment the variation of disease-associated alleles across the geographic regions.

Our results clearly indicate that there region/population-level specifics in the enrichment of disease-associated alleles, which could be linked to the disease prevalence. Moreover, we noticed a significant variation of disease predisposition background across the worldwide populations, in particular, for common diseases, such as diabetes, cancers, cardiovascular, and mental diseases. These observations confirm driving their multifactorial nature and involvement in multiple pathways their pathogenesis. It is worth to note that the low-frequency alleles associated with a disease in one population showed considerably high population levels frequency in another. We also observed a bias in present knowledge toward most prevalent diseases (like cancers, diabetes) as well as toward variants reported in Western World and few Asian and African populations. Future studies are required which focus more on so far understudied diseases and populations.

Further, our results raise the question of how the genetic risk in one population transfers into another one and it emphasizes the need for involving as much as possible populations into clinical genomics initiatives.

As an example of the understudied population, we focused on Armenians. Portraying of disease-related SNPs in Armenians demonstrated similarities with the Middle East, European and Central Asian populations. A more detailed analysis detected SNPs with specifically increased MAFs in Armenians compared with all other populations studied which indicates local disease prevalence in agreement with epidemiological data.

It is worth to notice a few limitations of our analysis. Population-level high MAFs does not necessarily indicate increased disease’s susceptibility or prevalence in a particular region. Alternatively, it can be also the result of long periods of “non-exposure” to the disease in a certain population. Next, our study neglected a large number of variations of disease-causing mutations, because they were not included neither in the array data used nor in available disease association catalogs. DNA sequencing will have more resolution in this respect, but there is presently no enough consistent data. Currently, there are several available datasets that contain exome or whole genome sequencing data from various population genetic and disease-specific studies, such as ExAC/gnomAD or 1000 Genomes which will enable studying population diversity based on larger number of samples. We have chosen HGDP for our methodical study because it provides a relative large population diversity which still exceeds that in the other datasets (51 in HGDP vs. 26 in 1000 Genomes, 10 in genomAD, and 17 in ExAC) and because of matched measuring platforms with SNP-data for Armenians. Future studies have to consider population diversity in Caucasus region and the surrounding areas and also ancient samples which become increasingly available for more detailed disease-risk profiling in space and time.

From a methodological point of view, our study demonstrates the power of machine learning and, particularly, of SOM portrayal or analyzing genomic data. This method possesses strong visualization capabilities by providing maps of the SNP-landscapes of the populations under study. They project the highly-multidimensional SNP-patterns strictly into two dimensions in contrast to principal component plots which still are multidimensional. Moreover and most importantly, the method generates a “SNP portrait” for each individual this way enabling the personalized evaluation of its SNP-patterns. These individual portraits can be used to generate mean portraits averaged over selected groups of individuals, e.g., of populations from selected geographic regions which then can be compared to identify common and different SNP patterns. The strong clustering capabilities of SOM deliver groups of SNPs showing similar profiles and application of enrichment techniques provide their functional and disease context. The method needs further development for applications to genomic data, for example, to include other genetic defects and to integrate and to visualize additional phenotypic information.

Overall, our novel approach extends the toolset employed of association and population genetic studies. The strength of SOM portrayal used here can be seen in the possibility of disentangling entire genetic variation landscape into functional clusters, which subsequently can be assigned to various features of the groups studied. This includes stratification of populations and identification of diseases associated variants.

## Conclusion

Our results clearly indicate that there is a great scope for further research in this area. There is a strong need to include non-Western populations in future studies that are clinically, geographically, and ethnically well-characterized.

## Author Contributions

AA and HB initiated the study. MN and AA performed calculations with contribution from SH, HL-W, and HB. MN, SH, AH, HL-W, HB, and AA contributed to results interpretation, manuscript writing, and approved the final manuscript.

## Conflict of Interest Statement

The authors declare that the research was conducted in the absence of any commercial or financial relationships that could be construed as a potential conflict of interest.

## Footnotes

1 http://www.hagsc.org/hgdp/

2 www.uniprot.org/docs/humsavar

3 https://www.ncbi.nlm.nih.gov/clinvar/

4 http://jjwanglab.org/gwasdb

5 http://www.disgenet.org/

## References

[B1] Abraham G. Inouye M. (2015). Genomic risk prediction of complex human disease and its clinical application. *Curr. Opin. Genet. Dev.* 33 10–16. 10.1016/j.gde.2015.06.005 26210231

[B2] Andreasyan D. Bazarchyan A. Simonyan S. Muradyan G. Simonyan A. Matevosyan M. (2017). *Health and Health Care Yearbook, RA 2017*. Yerevan, Armenia: National Institute of Health after academician S. Avdalbekyan. ISBN: 978-9939-9156-8-5.

[B3] Arakelyan A. Nersisyan L. Poghosyan D. Khondkaryan L. Hakobyan A. Löffler-Wirth H. (2017). Autoimmunity and autoinflammation: a systems view on signaling pathway dysregulation profiles. *PLoS One* 12:e0187572. 10.1371/journal.pone.0187572 29099860 PMC5669448

[B4] Bailey R. L. West K. P. Black R. E. (2015). The epidemiology of global micronutrient deficiencies. *Ann. Nutr. Metab.* 66 22–33. 10.1159/000371618 26045325

[B5] Baxter A. J. Scott K. M. Vos T. Whiteford H. A. (2013). Global prevalence of anxiety disorders: a systematic review and meta-regression. *Psychol. Med.* 43 897–910. 10.1017/S003329171200147X 22781489

[B6] Benziger C. P. Roth G. A. Moran A. E. (2016). The global burden of disease study and the preventable burden of NCD. *Glob. Heart* 11 393–397. 10.1016/j.gheart.2016.10.024 27938824

[B7] Bernatzky G. Kullich W. Aglas F. Ausserwinkler M. Likar R. Pipam W. (2009). Electromagnetic fields in patients with chronic low-back pain: a double-blind randomized placebo-controlled two-centre study. *Schweiz. Z. Ganzheitsmed.* 21 149–156. 10.1159/000287221

[B8] Billingsley K. J. Bandres-Ciga S. Saez-Atienzar S. Singleton A. B. (2018). Genetic risk factors in Parkinson’s disease. *Cell Tissue Res.* 373 9–20. 10.1007/s00441-018-2817-y 29536161 PMC6201690

[B9] Binder H. Wirth H. (2014). “Analysis of large-scale omic data using self organizing maps,” in *Encyclopedia of Information Science and Technology* 3rd Edn. ed. Khosrow-Pour M. (Pennsylvania: IGI Global) 1642–1653. 10.4018/978-1-4666-5888-2.ch157

[B10] Binder H. Wirth H. Arakelyan A. Lembcke K. Tiys E. S. Ivanisenko V. A. (2014). Time-course human urine proteomics in space-flight simulation experiments. *BMC Genomics* 15(Suppl. 12):S2. 10.1186/1471-2164-15-12-S2 25563515 PMC4303941

[B11] Chen G. Ramos E. Adeyemo A. Shriner D. Zhou J. Doumatey A. P. (2012). UGT1A1 is a major locus influencing bilirubin levels in African Americans. *Eur. J. Hum. Genet.* 20 463–468. 10.1038/ejhg.2011.206 22085899 PMC3306855

[B12] Colhoun H. M. McKeigue P. M. Smith G. D. (2003). Problems of reporting genetic associations with complex outcomes. *Lancet* 361 865–872. 10.1016/S0140-6736(03)12715-8 12642066

[B13] Cox A. J. Ng M. C. Y. Xu J. Langefeld C. D. Koch K. L. Dawson P. A. (2013). Association of SNPs in the UGT1A gene cluster with total bilirubin and mortality in the diabetes heart study. *Atherosclerosis* 229 155–160. 10.1016/j.atherosclerosis.2013.04.008 23642732 PMC3691283

[B14] Engelman C. D. Meyers K. J. Ziegler J. T. Taylor K. D. Palmer N. D. Haffner S. M. (2010). Genome-wide association study of vitamin D concentrations in Hispanic Americans: the IRAS family study. *J. Steroid Biochem. Mol. Biol.* 122 186–192. 10.1016/j.jsbmb.2010.06.013 20600896 PMC2949505

[B15] Foley C. Corvin A. Nakagome S. (2017). Genetics of schizophrenia: ready to translate? *Curr. Psychiatry Rep.* 19:61. 10.1007/s11920-017-0807-5 28741255

[B16] Foreman K. J. Barac A. Williams H. C. Sufiyan M. B. Safiri S. Sinke A. H. (2018). Global, regional, and national cancer incidence, mortality, years of life lost, years lived with disability, and disability-adjusted life-years for 29 cancer groups, 1990 to 2016. *JAMA Oncol.* 4 1553–1568. 10.1001/jamaoncol.2018.2706 29860482 PMC6248091

[B17] Giri M. Zhang M. Lü Y. (2016). Genes associated with Alzheimer’s disease: an overview and current status. *Clin. Interv. Aging* 11 665–681. 10.2147/CIA.S105769 27274215 PMC4876682

[B18] Guino E. Iniesta R. Sole X. Moreno V. Valls J. (2006). SNPStats: a web tool for the analysis of association studies. *Bioinformatics* 22 1928–1929. 10.1093/bioinformatics/btl268 16720584

[B19] Haber M. Mezzavilla M. Xue Y. Comas D. Gasparini P. Zalloua P. (2016). Genetic evidence for an origin of the Armenians from Bronze Age mixing of multiple populations. *Eur. J. Hum. Genet.* 24 931–936. 10.1038/ejhg.2015.206 26486470 PMC4820045

[B20] Habib S. H. Saha S. (2010). Burden of non-communicable disease: global overview. *Diabetes Metab. Syndr. Clin. Res. Rev.* 4 41–47. 10.1016/J.DSX.2008.04.005

[B21] Hopp L. Loeffler-Wirth H. Nersisyan L. Arakelyan A. Binder H. (2018). Footprints of sepsis framed within community acquired pneumonia in the blood transcriptome. *Front. Immunol.* 9:1620. 10.3389/fimmu.2018.01620 30065722 PMC6056630

[B22] Hopp L. Nersisyan L. Löffler-Wirth H. Arakelyan A. Binder H. (2015). Epigenetic heterogeneity of B-cell lymphoma: chromatin modifiers. *Genes* 6 1076–1112. 10.3390/genes6041076 26506391 PMC4690029

[B23] Hovhannisyan A. Khachatryan Z. Haber M. Hrechdakian P. Karafet T. Zalloua P. (2014). Different waves and directions of Neolithic migrations in the Armenian Highland. *Investig. Genet.* 5:15. 10.1186/s13323-014-0015-6 25452838 PMC4249771

[B24] Jankovic I. Huang L. Boehnke M. Rosenberg N. A. Szpiech Z. A. Jewett E. M. (2010). Genome-wide association studies in diverse populations. *Nat. Rev. Genet.* 11 356–366. 10.1038/nrg2760 20395969 PMC3079573

[B25] Kakkar R. (2016). Rising burden of diabetes-public health challenges & way out. *Nepal J. Epidemiol.* 6 557–559. 10.3126/nje.v6i2.15160 27774342 PMC5073171

[B26] Kim M. S. Patel K. P. Teng A. K. Berens A. J. Lachance J. (2018). Genetic disease risks can be misestimated across global populations. *Genome Biol.* 19:179. 10.1186/s13059-018-1561-7 30424772 PMC6234640

[B27] Kim Y. S. Kang T.-W. Kim K.-K. Ju H. Lee S. Jeon Y.-J. (2010). Genome-wide association of serum bilirubin levels in Korean population. *Hum. Mol. Genet.* 19 3672–3678. 10.1093/hmg/ddq281 20639394 PMC2928134

[B28] Kristiansson K. Naukkarinen J. Peltonen L. (2008). Isolated populations and complex disease gene identification. *Genome Biol.* 9:109. 10.1186/gb-2008-9-8-109 18771588 PMC2575505

[B29] Lachance J. (2010). Disease-associated alleles in genome-wide association studies are enriched for derived low frequency alleles relative to HapMap and neutral expectations. *BMC Med. Genomics* 3:57. 10.1186/1755-8794-3-57 21143973 PMC3017004

[B30] Lau L. T. Yee A. Fok M. Lam C. W. K. He D. Lau J. Y. N. (2018). Population-wide genetic risk prediction of complex diseases: a pilot feasibility study in Macau population for precision public healthcare planning. *Sci. Rep.* 8:1853. 10.1038/s41598-017-19017-y 29382849 PMC5789865

[B31] Lennikov A. Alekberova Z. Goloeva R. Kitaichi N. Denisov L. Namba K. (2015). Single center study on ethnic and clinical features of Behcet’s disease in Moscow, Russia. *Clin. Rheumatol.* 34 321–327. 10.1007/s10067-013-2442-9 24322831

[B32] Leonardo N. M. McNeil J. (2015). Behcet’s disease: is there geographical variation? A review far from the Silk Road. *Int. J. Rheumatol.* 2015 1–7. 10.1155/2015/945262 26798344 PMC4698787

[B33] Li A. Meyre D. (2013). Challenges in reproducibility of genetic association studies: lessons learned from the obesity field. *Int. J. Obes.* 37 559–567. 10.1038/ijo.2012.82 22584455

[B34] Löffler-Wirth H. Kalcher M. Binder H. (2015). OposSOM: R-package for high-dimensional portraying of genome-wide expression landscapes on bioconductor. *Bioinformatics* 31 3225–3227. 10.1093/bioinformatics/btv342 26063839

[B35] Memon N. Weinberger B. I. Hegyi T. Aleksunes L. M. (2016). Inherited disorders of bilirubin clearance. *Pediatr. Res.* 79 378–386. 10.1038/pr.2015.247 26595536 PMC4821713

[B36] Müller U. (2006). “Cell adhesion molecules and human disorders,” in *eLS* Chichester: John Wiley & Sons Ltd 10.1038/npg.els.0005169

[B37] Nikoghosyan M. Hakobyan S. Hovhannisyan A. Loffler-Wirth H. Binder H. Arakelyan A. (2018). Population Level Assessment of Disease Associated Genetic Variants With Emphasis on Armenians - A Machine Learning Approach (Supplementary Datasets). 10.5281/ZENODO.1484829 PMC6498285 31105750

[B38] Obenchain V. Lawrence M. Carey V. Gogarten S. Shannon P. Morgan M. (2014). VariantAnnotation: a Bioconductor package for exploration and annotation of genetic variants. *Bioinformatics* 30 2076–2078. 10.1093/bioinformatics/btu168 24681907 PMC4080743

[B39] Oke W. A. Khulief Y. A. (2016). Vibration analysis of composite pipes using the finite element method with B-spline wavelets. *J. Mech. Sci. Technol.* 30 623–635. 10.1007/s12206-016-0116-7

[B40] Prentice A. (2008). Vitamin D deficiency: a global perspective. *Nutr. Rev.* 66(10 Suppl. 2) S153–S164. 10.1111/j.1753-4887.2008.00100.x 18844843

[B41] Prentice A. Schoenmakers I. Jones K. S. Jarjou L. M. A. Goldberg G. R. (2009). Vitamin D deficiency and its health consequences in Africa. *Clin. Rev. Bone Miner. Metab.* 7 94–106. 10.1007/s12018-009-9038-6 25110467 PMC4126271

[B42] Price A. L. Spencer C. C. A. Donnelly P. (2015). Progress and promise in understanding the genetic basis of common diseases. *Proc. R. Soc. B Biol. Sci.* 282:20151684. 10.1098/rspb.2015.1684 26702037 PMC4707742

[B43] Pritchard J. K. (2001). Are rare variants responsible for susceptibility to complex diseases? *Am. J. Hum. Genet.* 69 124–137. 10.1086/321272 11404818 PMC1226027

[B44] Qi Y. Zheng Y. Li Z. Xiong L. (2017). Progress in genetic studies of tourette’s syndrome. *Brain Sci.* 7:E134. 10.3390/brainsci7100134 29053637 PMC5664061

[B45] Ramos R. G. Olden K. (2008). Gene-environment interactions in the development of complex disease phenotypes. *Int. J. Environ. Res. Public Health* 5 4–11. 10.3390/ijerph5010004 18441400 PMC3684407

[B46] Ran P. Zhao J. Cai C. Mo Z. Zhu J. Jiang F. (2011). Analysis of the specific pathways and networks of prostate cancer for gene expression profiles in the Chinese population. *Med. Oncol.* 29 1972–1984. 10.1007/s12032-011-0088-5 22038724

[B47] Reich D. E. Lander E. S. (2001). On the allelic spectrum of human disease. *Trends Genet.* 17 502–510. 10.1016/S0168-9525(01)02410-6 11525833

[B48] Rice J. P. Merikangas K. R. Hunter D. J. Abecasis G. Daly M. Roberts J. (2007). Replicating genotype–phenotype associations. *Nature* 447 655–660. 10.1038/447655a 17554299

[B49] Rosenberg N. A. (2006). Standardized subsets of the HGDP-CEPH Human Genome Diversity Cell Line Panel, accounting for atypical and duplicated samples and pairs of close relatives. *Ann. Hum. Genet.* 70 841–847. 10.1111/j.1469-1809.2006.00285.x 17044859

[B50] Saberi P. McKenzie J. Emmett E. (2012). P-117: field survey of health complaints of pennsylvania residents in marcellus shale regions. *Epidemiology* 23:1 10.1097/01.ede.0000417122.87403.c8 PMC4078593 25003172

[B51] Schriml L. M. Arze C. Nadendla S. Chang Y. W. W. Mazaitis M. Felix V. (2012). Disease ontology: a backbone for disease semantic integration. *Nucleic Acids Res.* 40 D940–D946. 10.1093/nar/gkr972 22080554 PMC3245088

[B52] Steel Z. Marnane C. Iranpour C. Chey T. Jackson J. W. Patel V. (2014). The global prevalence of common mental disorders: a systematic review and meta-analysis 1980-2013. *Int. J. Epidemiol.* 43 476–493. 10.1093/ije/dyu038 24648481 PMC3997379

[B53] Sud A. Kinnersley B. Houlston R. S. (2017). Genome-wide association studies of cancer: current insights and future perspectives. *Nat. Rev. Cancer* 17 692–704. 10.1038/nrc.2017.82 29026206

[B54] Tataryan K. (2012). Some data about Alzheimer’s disease. *Alzheimers Dement.* 8:P315 10.1016/j.jalz.2012.05.867

[B55] Wang J. Vasaikar S. Shi Z. Greer M. Zhang B. (2017). WebGestalt 2017: a more comprehensive, powerful, flexible and interactive gene set enrichment analysis toolkit. *Nucleic Acids Res.* 45 W130–W137. 10.1093/nar/gkx356 28472511 PMC5570149

[B56] WHO (2013). *Nutrition, Physical Activity and Obesity*.

[B57] Wirth H. Löffler M. von Bergen M. Binder H. (2011). Expression cartography of human tissues using self organizing maps. *BMC Bioinformatics* 12:306. 10.1186/1471-2105-12-306 21794127 PMC3161046

[B58] Yepiskoposyan L. Hovhannisyan A. Khachatryan Z. (2016). Genetic structure of the Armenian population. *Arch. Immunol. Ther. Exp.* 64 113–116. 10.1007/s00005-016-0431-9 28083603

[B59] Zheng F. Wei L. Zhao L. Ni F. (2018). Pathway network analysis of complex diseases based on multiple biological networks. *Biomed Res. Int.* 2018:5670210. 10.1155/2018/5670210 30151386 PMC6091292

