# Supplementary material for: Population Levels Assessment of the Distribution of Disease-Associated Variants With Emphasis on Armenians – A Machine Learning Approach
Source: Front Genet. 2019 Apr 26;10:394. doi: 10.3389/fgene.2019.00394 (PMC6498285; doi:10.3389/fgene.2019.00394)
Supplement: Supplementary file 1 [file Data_Sheet_1.PDF]

Supplementary Material

Population levels assessment of the distribution of disease associated variants - a machine learning approach

Mariya Nikoghosyan[1,2], Siras Hakobyan[2], Anahit Hovhannisyan[3], Henry Loeffler-Wirth[4], Hans Binder[4+], Arsen Arakelyan[1,2*+]

[1]Russian - Armenian University, Institute of Biomedicine and Pharmacy, Yerevan, Armenia

[2]Research Group of Bioinformatics, Institute of Molecular Biology NAS RA, Yerevan, Armenia

[3]Laboratory of Ethnogenomics, Institute of Molecular Biology NAS RA, Yerevan, Armenia

[4]Interdisciplinary Centre for Bioinformatics, University of Leipzig, Leipzig, Germany

*** Correspondence:**
Arsen Arakelyan,
arsen.arakelyan@rau.am

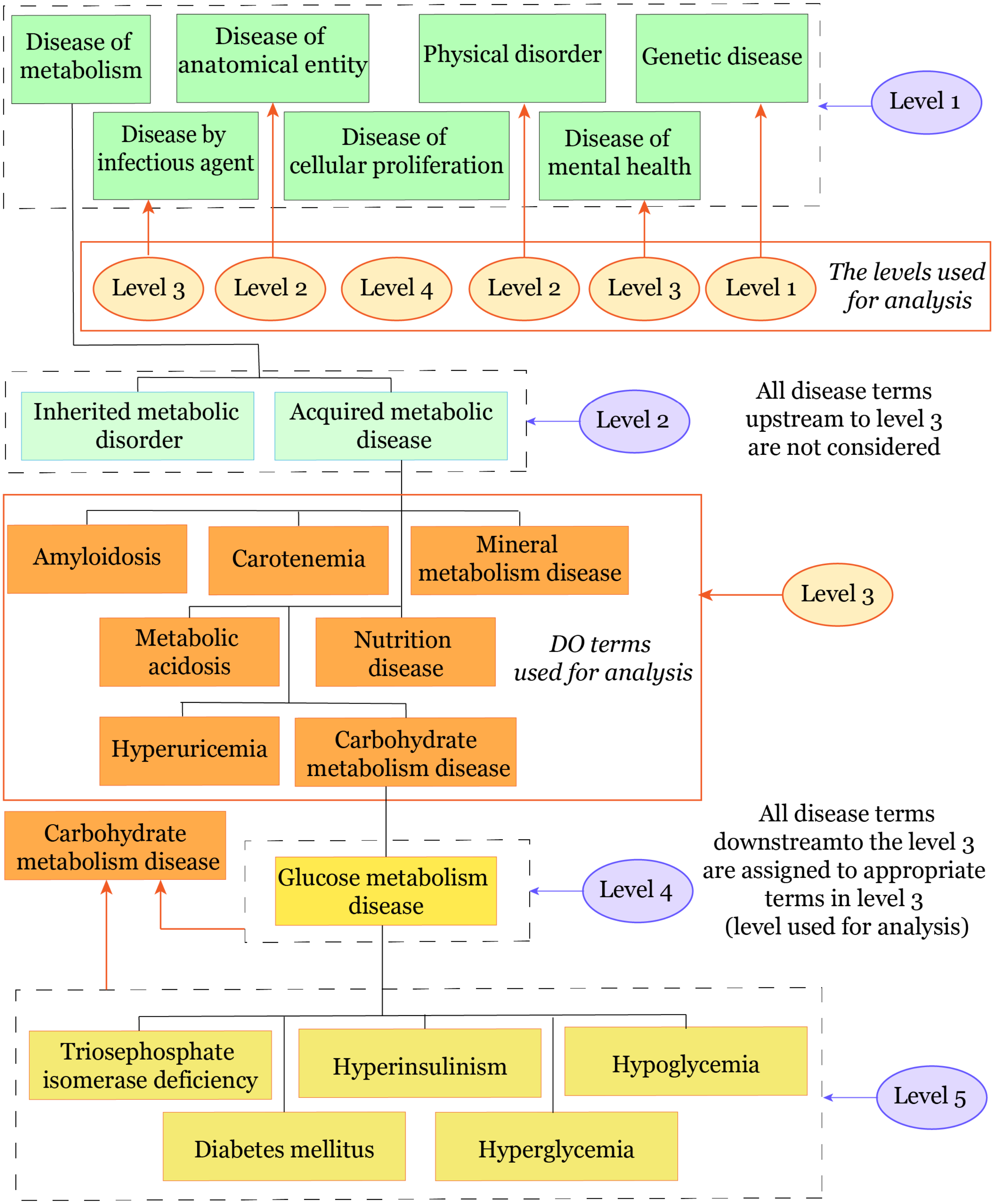

Supplementary Figure 1. The scheme represents DO terms mapping. For each DO term in level 1 (in the top) the most appropriate level is selected. All terms downstream to the selected level are mapped into the appropriate term in selected level (e.g. all term in level 4 and 5 for Disease of

metabolism are mapped into appropriate terms in level 3). On the other hand, all term upstream to the selected level are not considered.

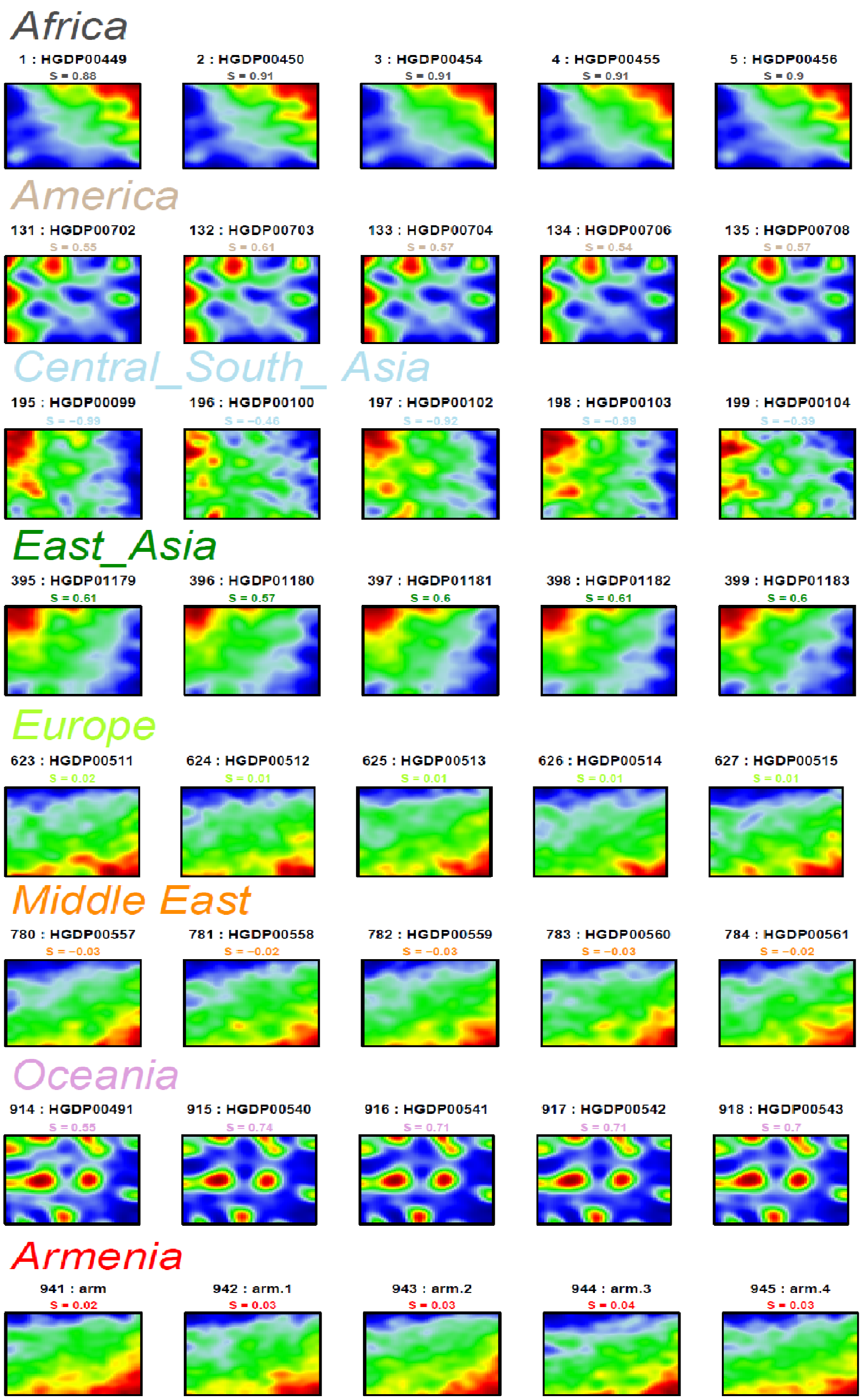

Supplementary Figure 2. “Personal” SNP portraits for selected individuals from 8 geographic regions. Complete personal portraits are available in the accompanying dataset (Nikoghosyan et al., 2018).

A. Three dimensional ICA plot B. 2D projections

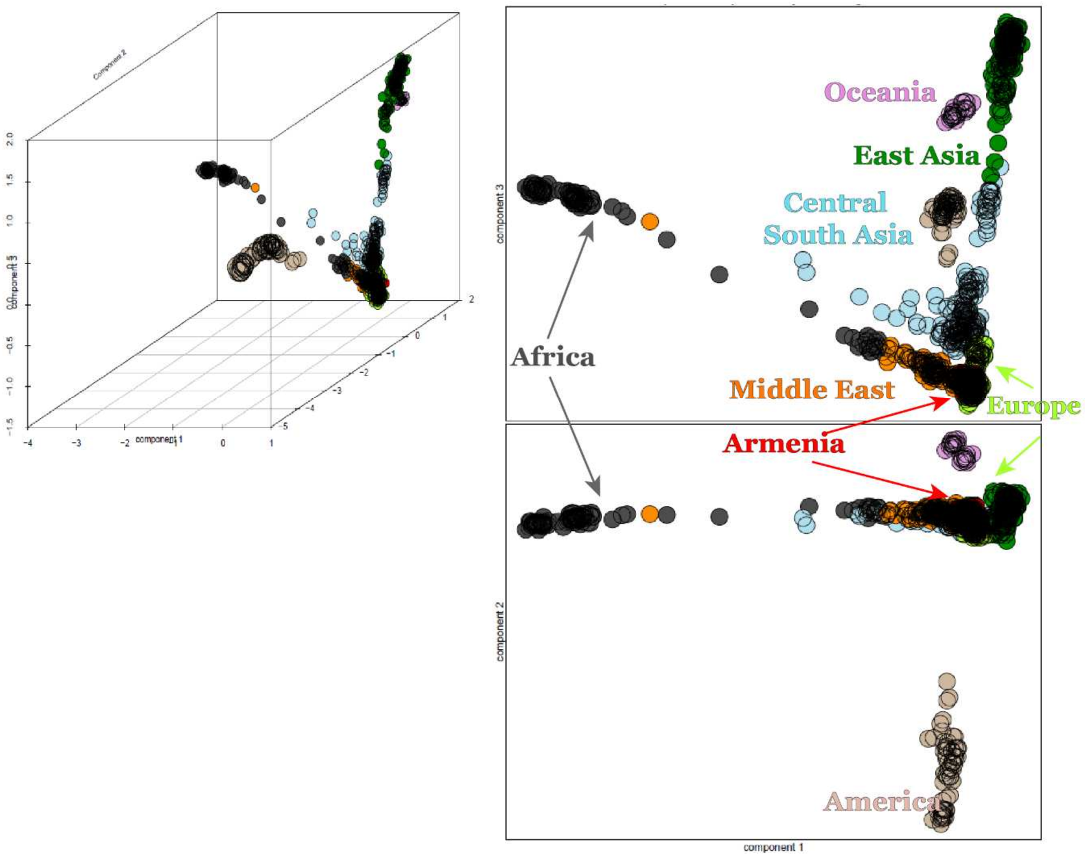

Supplementary Figure S3. Independent component plot (ICA) of the SNP SOM portraits of the individuals studied. The plot shows the first three ICS-components (IC1 – IC3) as dimensions. The SNP landscapes of nearly all populations arrange into the plane spanned by the IC1 and IC3 dimensions. Only Americans and, to a less degree Oceanians, are located out of this plane into dimension IC2. Note that ICA plots the distances between the samples in scale with non-Gaussian (i.e. non-random) differences between their SNP landscapes. Accordingly, one identifies separate clusters occupied predominantly by Africans, East Asians, Americans, Oceanians and a joint cluster collecting people from Europe, the Middle East and Central Asia. Independent component analysis (ICA) was applied to the SOM meta-genes using the R package "fastICA" [Ref1]. It distributes the samples in the space spanned by the components of minimum mutual statistical dependence. These components point along the directions of maximum information content in the data which is estimated by their deviation from a (non-informative) Normal Distribution [Ref2]. ICA was based on covariance matrix calculated in terms of Pearson correlation coefficients between all meta-genes from any two samples.

Ref1: Hyvärinen A, Oja E. Independent component analysis: algorithms and applications. Neural Netw 2000; 13:411-30; PMID:10946390; http://dx.doi. org/10.1016/S0893-6080(00)00026-5

Ref2: Liebermeister W. Linear modes of gene expression determined by independent component analysis. Bioinformatics 2002; 18:51-60; PMID:11836211; http://dx.doi.org/10.1093/bioinformatics/18.1.51

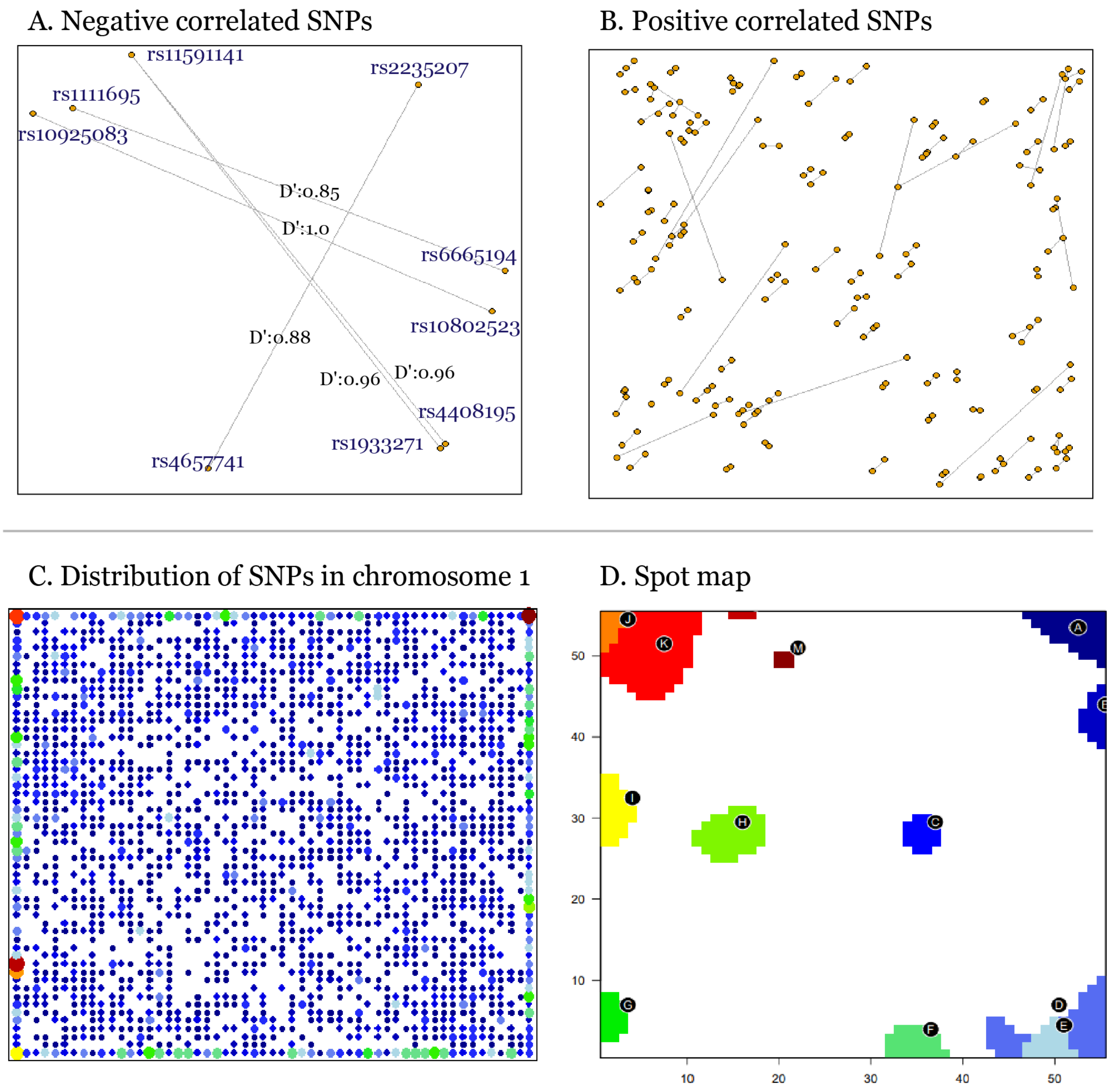

Supplementary Figure S4. Visualization of Linkage Disequilibrium on SOM generated SNP portraits. For visualization of LD SNPs located in chromosome 1 were selected from the studied dataset. The SOM algorithm tries to allocate SNPs with correlated profiles in close proximity (or in the same) cluster, while SNPs with anti-correlated profiles are positioned in furthest regions of the SNP portrait. (A) SNPs that are in LD located in distant clusters for negatively associated alleles, (B) SNPs that are in LD mostly located in same clusters for positively associated alleles. For visualization LD D' > 0.85 was selected with a threshold (C) Background distribution of all SNPs on chromosome 1 present in the studied dataset mapped on SOM global portrait. The color represents the density of SNPs in that position (from light blue to red, from low to high density, respectively). (D) The location of overexpression spots on the global portrait.

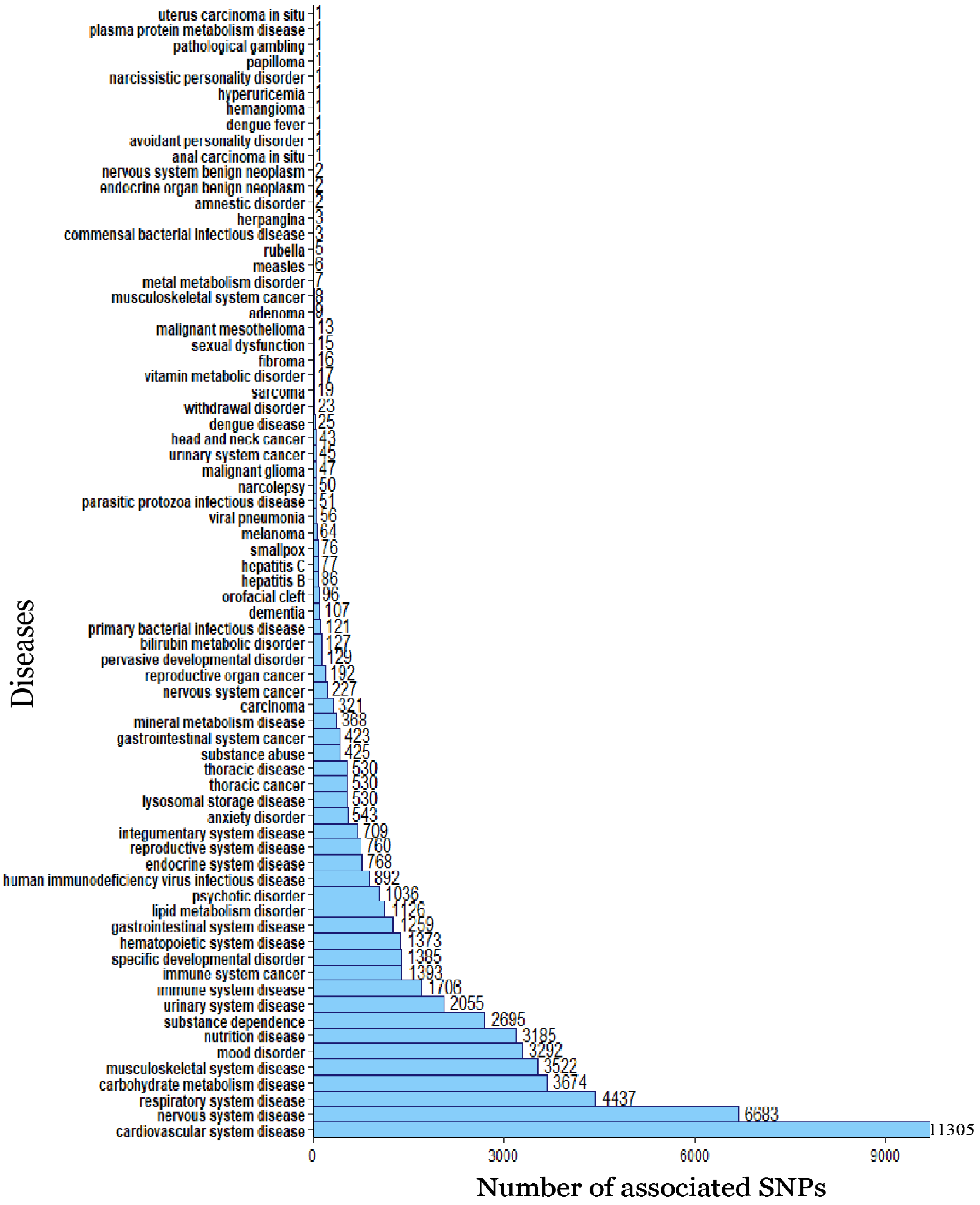

Supplementary Figure 5. Global disease-SNP distribution in input data. Number of SNPs associated with each disease.

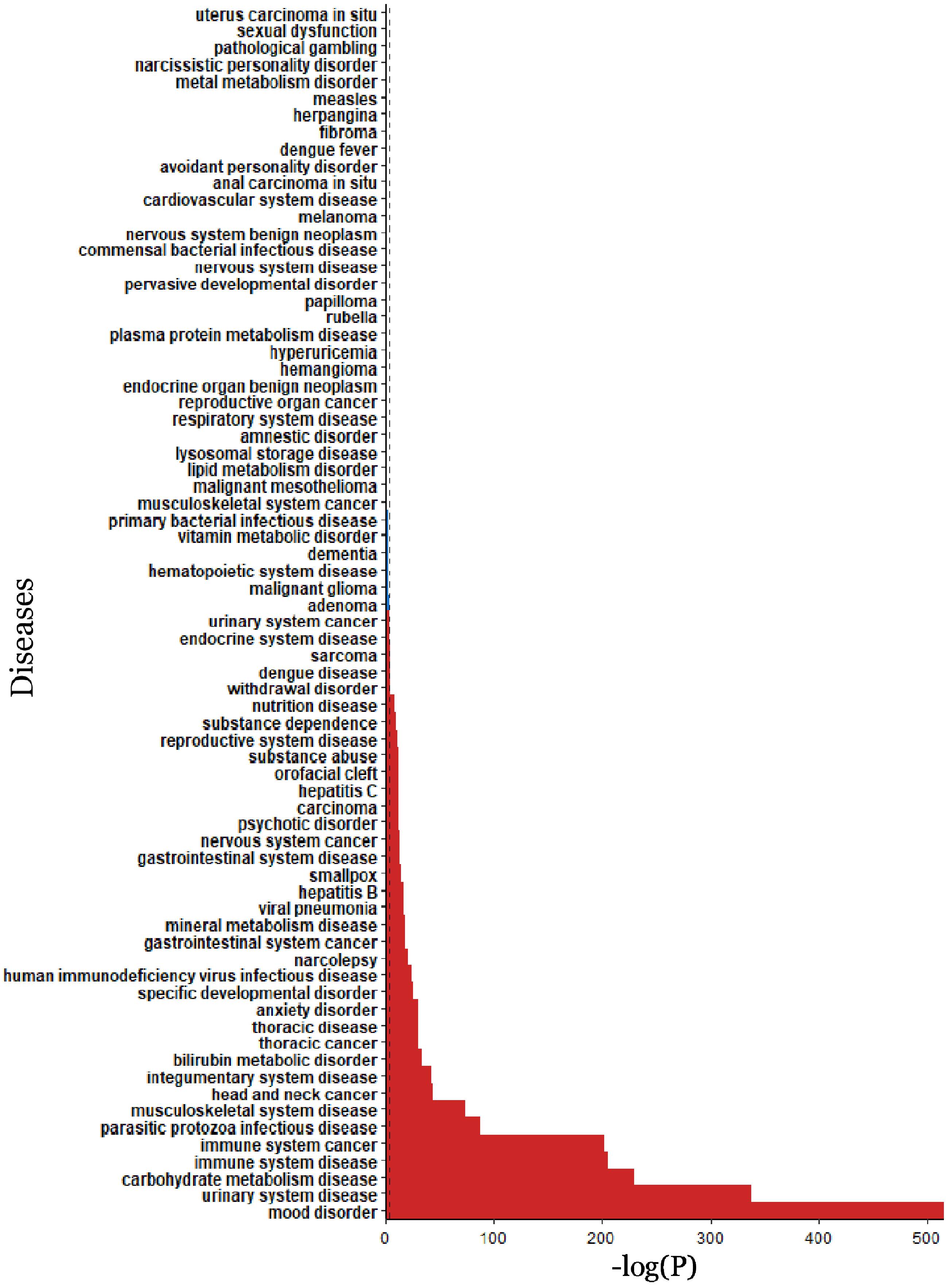
Diseases
uterus carcinoma in situ
sexual dysfunction
pathological gambling
narcissistic personality disorder
metal metabolism disorder
measles
herpangina
fibroma
dengue fever
avoidant personality disorder
anal carcinoma in situ
cardiovascular system disease
melanoma
nervous system benign neoplasm
commensal bacterial infectious disease
nervous system disease
pervasive developmental disorder
papilloma
rubella
plasma protein metabolism disease
hyperuricemia
hemangioma
endocrine organ benign neoplasm
reproductive organ cancer
respiratory system disease
amnestic disorder
lysosomal storage disease
lipid metabolism disorder
malignant mesothelioma
musculoskeletal system cancer
primary bacterial infectious disease
vitamin metabolic disorder
dementia
hematopoietic system disease
malignant glioma
adenoma
urinary system cancer
endocrine system disease
sarcoma
dengue disease
withdrawal disorder
nutrition disease
substance dependence
reproductive system disease
substance abuse
orofacial cleft
hepatitis C
carcinoma
psychotic disorder
nervous system cancer
gastrointestinal system disease
smallpox
hepatitis B
viral pneumonia
mineral metabolism disease
gastrointestinal system cancer
narcolepsy
human immunodeficiency virus infectious disease
specific developmental disorder
anxiety disorder
thoracic disease
thoracic cancer
bilirubin metabolic disorder
integumentary system disease
head and neck cancer
musculoskeletal system disease
parasitic protozoa infectious disease
immune system cancer
immune system disease
carbohydrate metabolism disease
urinary system disease
mood disorder
0
100
200
300
400
500
-log(P)

Supplementary Figure 6. Global disease-SNP distribution in input data**.** Significance of disease SNP enrichment.

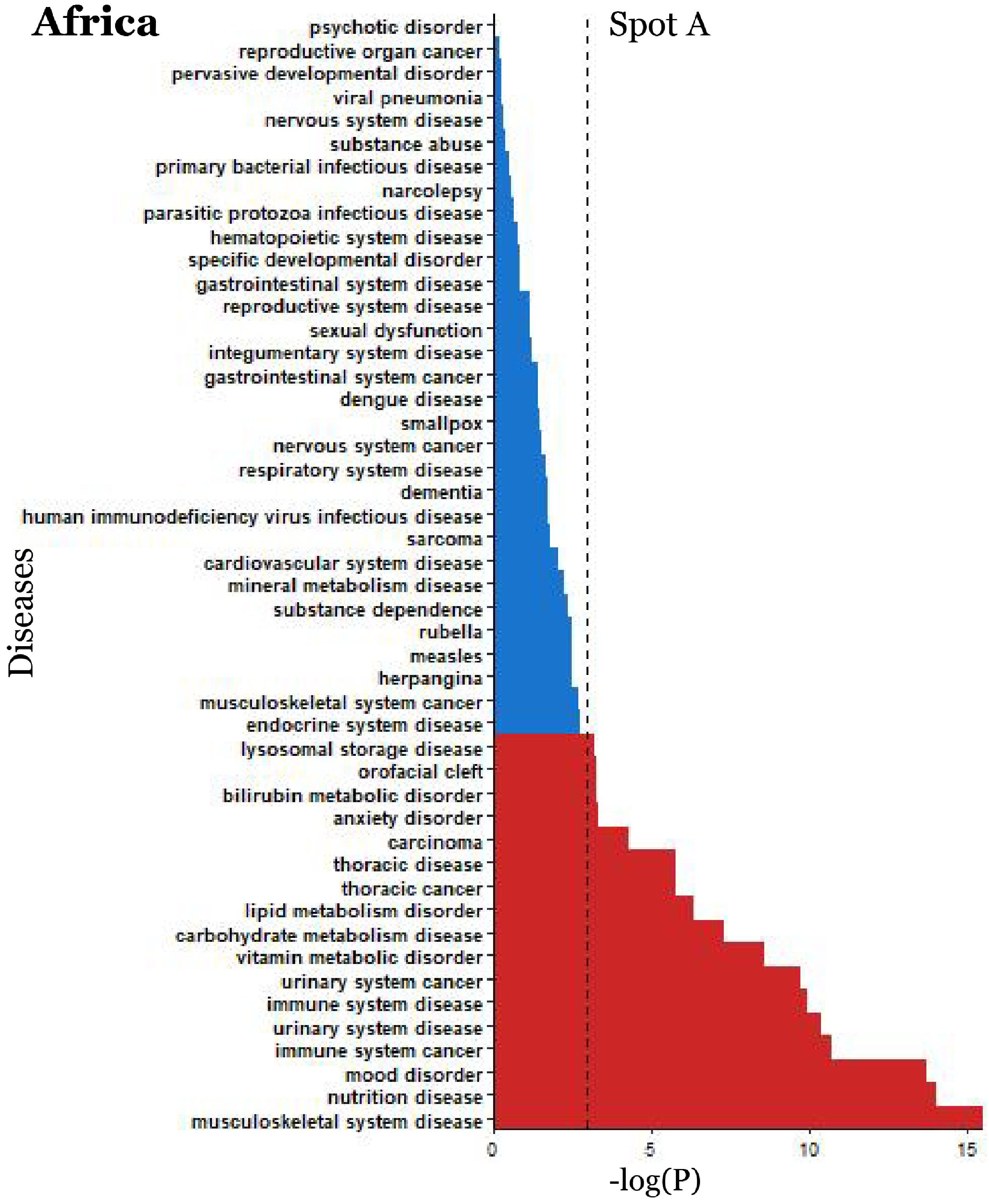

Supplementary Figure 7. Disease enrichment is spots A.

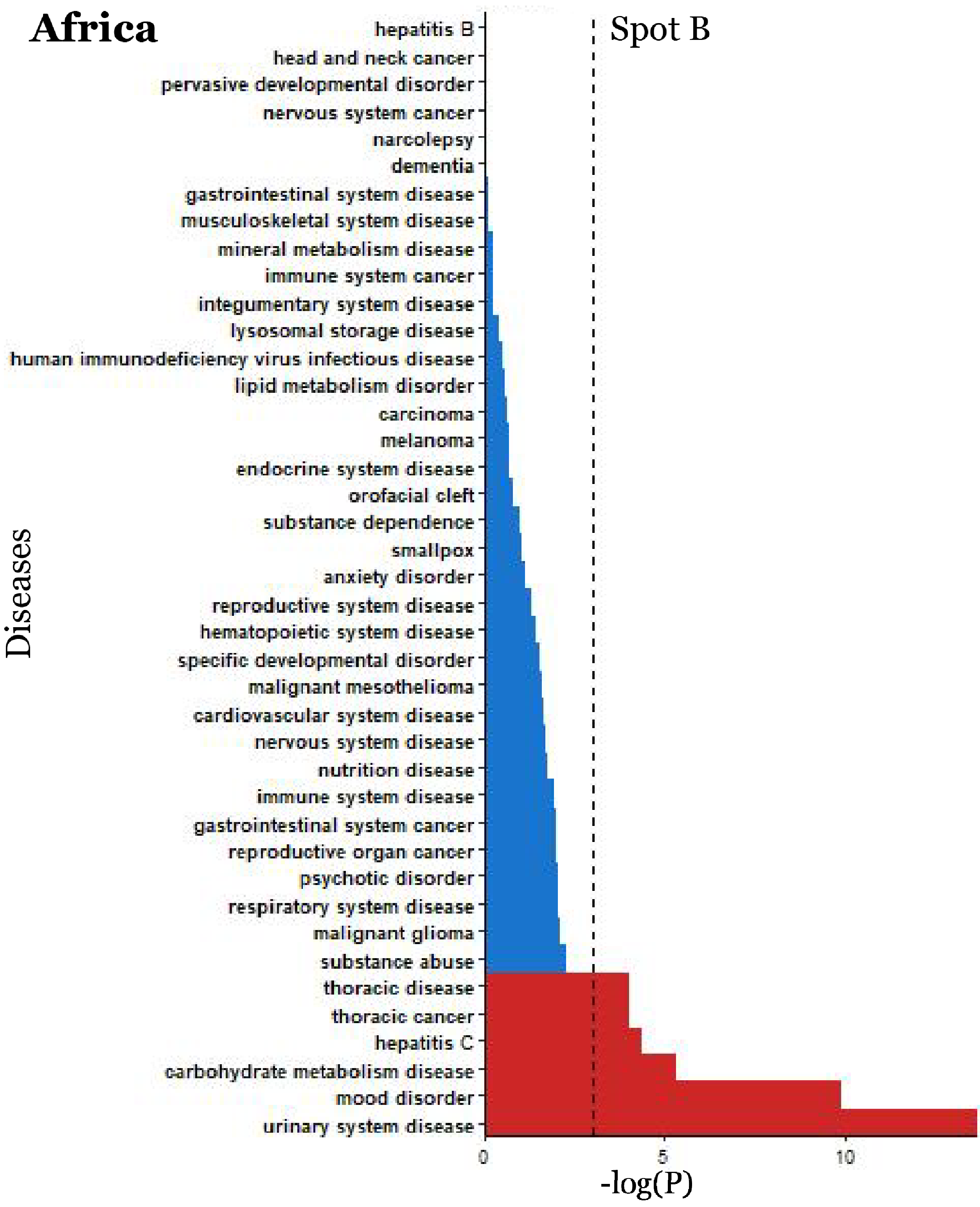

Supplementary Figure 8. Disease enrichment is spots B

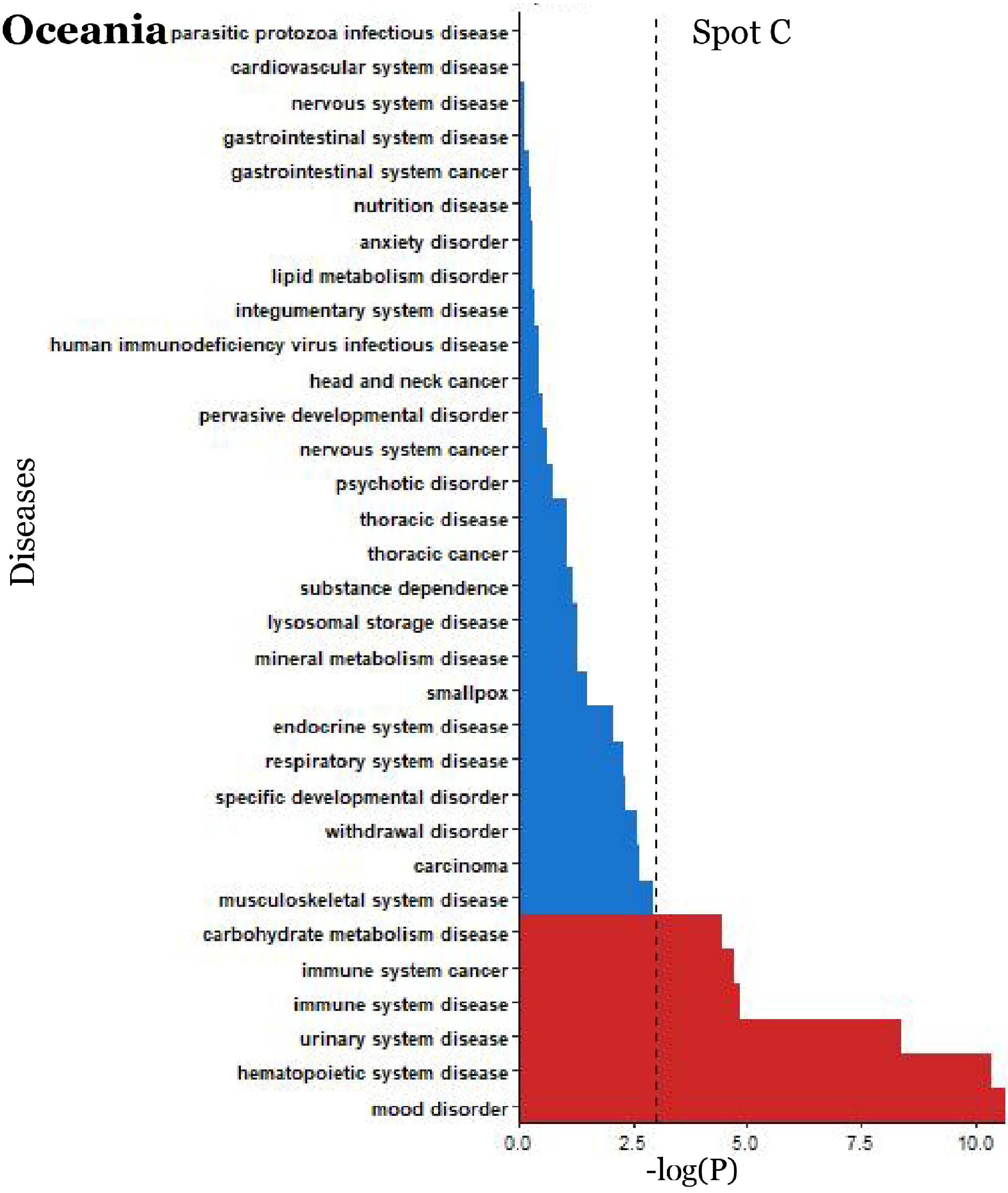

Supplementary Figure 9. Disease enrichment is the spots C.

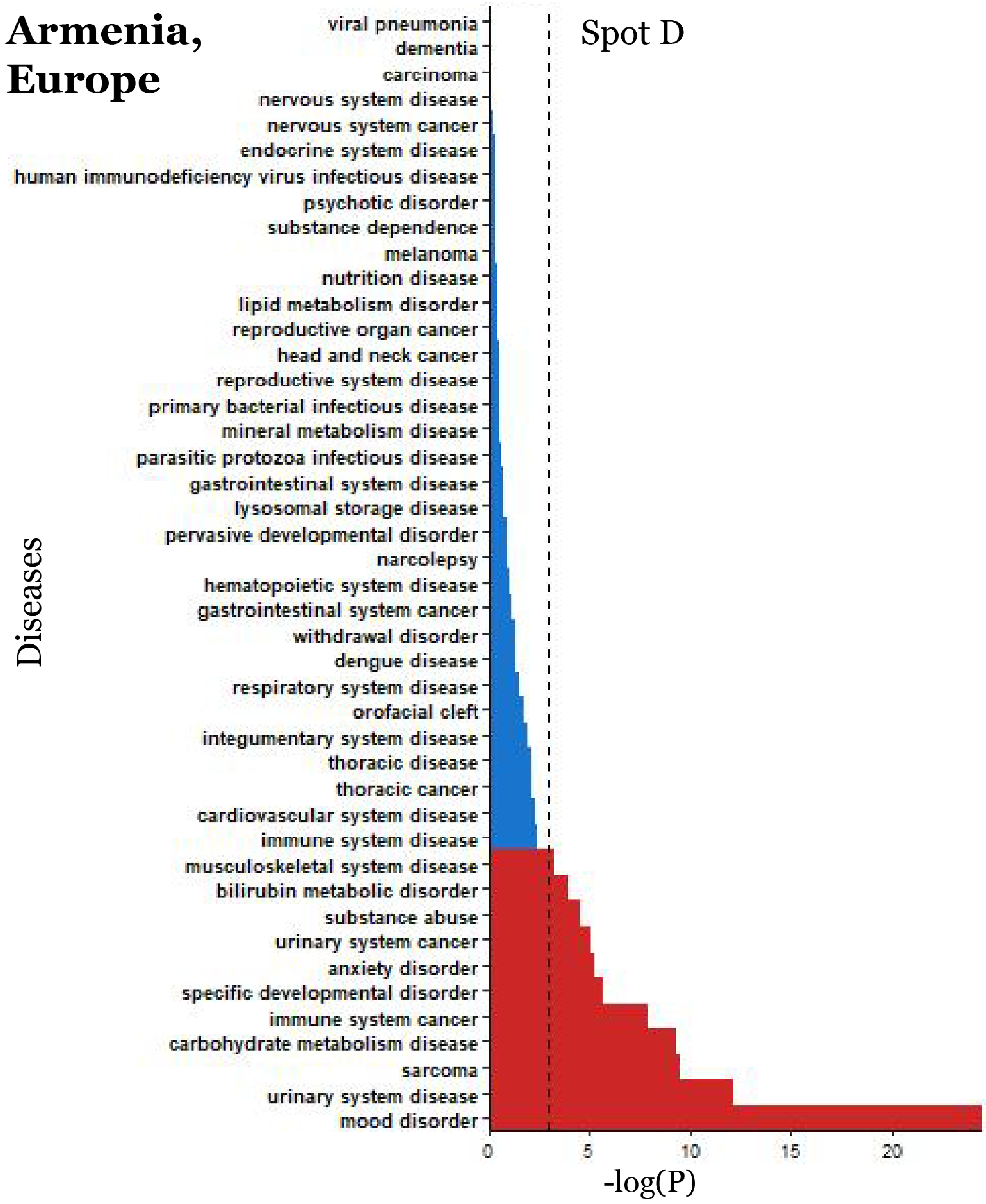

Supplementary Figure 10. Disease enrichment is the spots D.

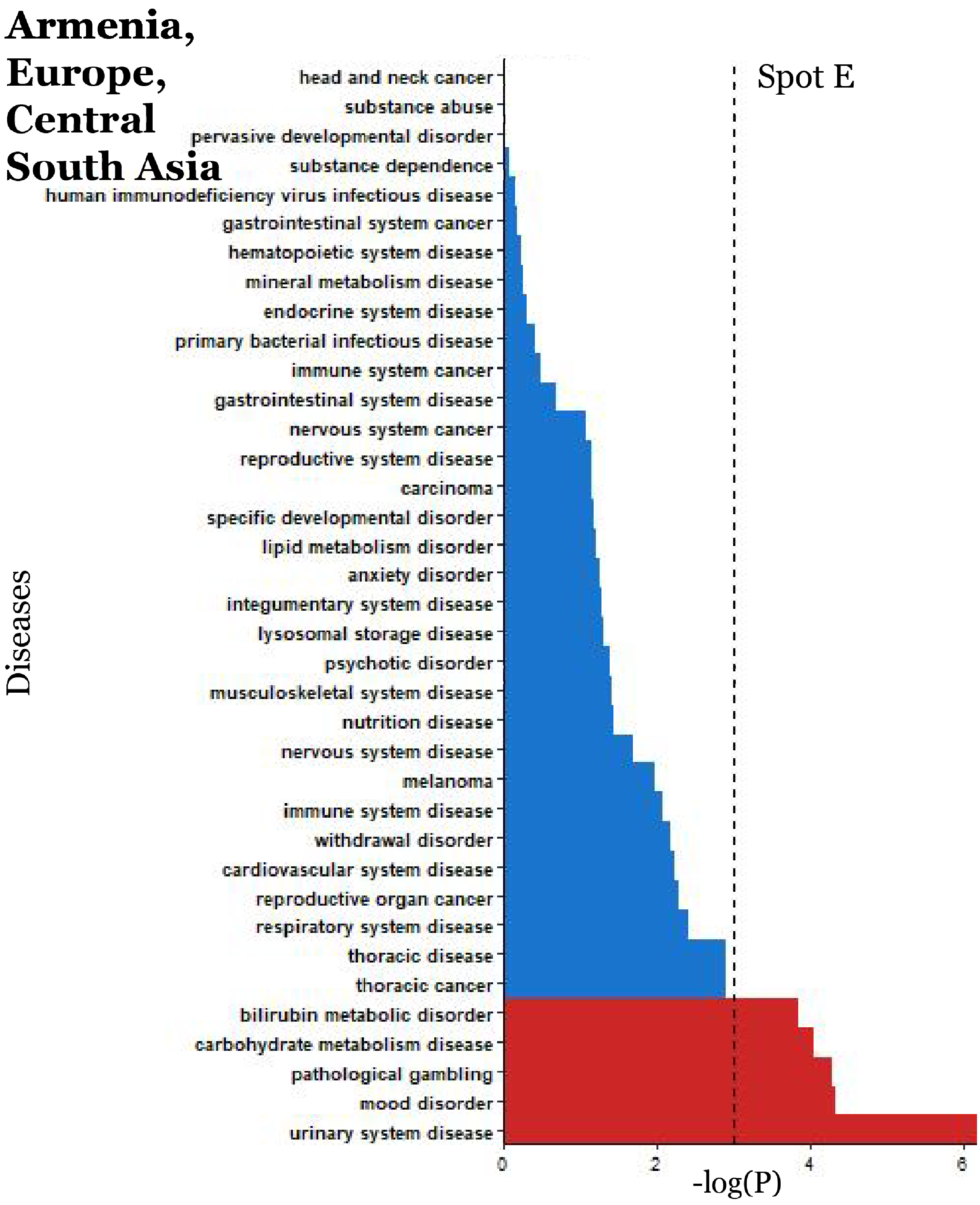

Supplementary Figure 11. Disease enrichment is the spots E.

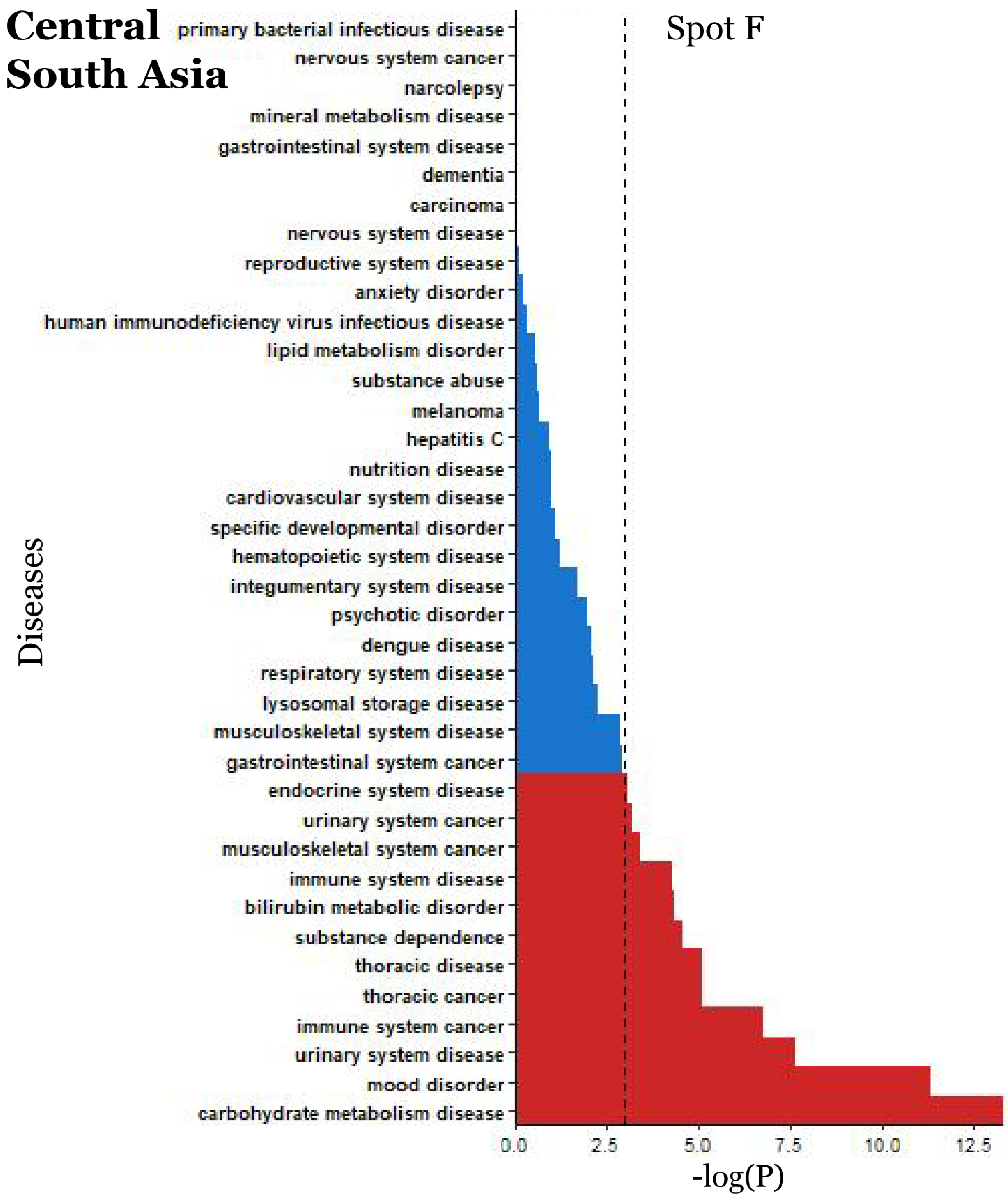

Supplementary Figure 12. Disease enrichment is the spots F.

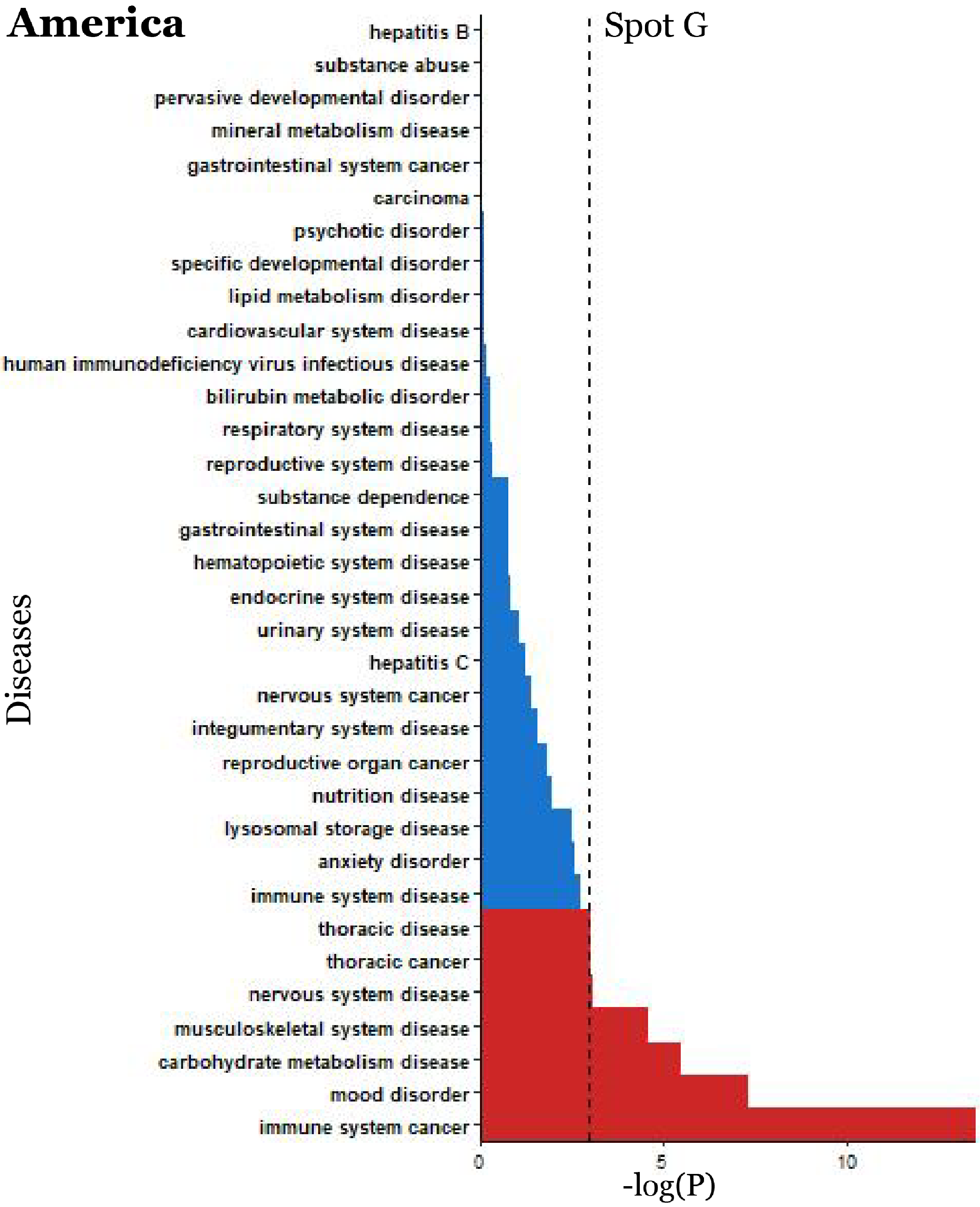

Supplementary Figure 13. Disease enrichment is the spots G.

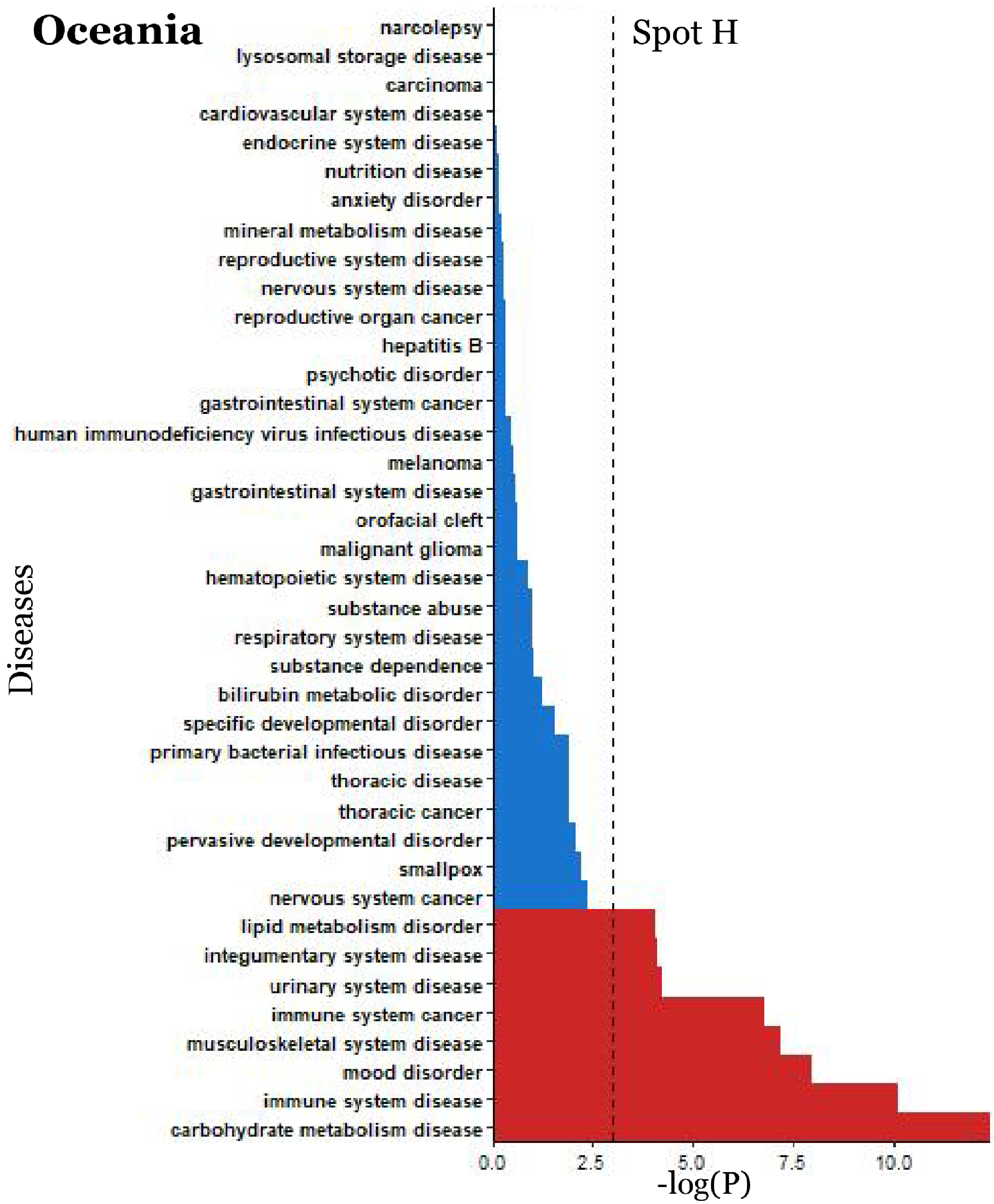

Supplementary Figure 14. Disease enrichment is the spots H.

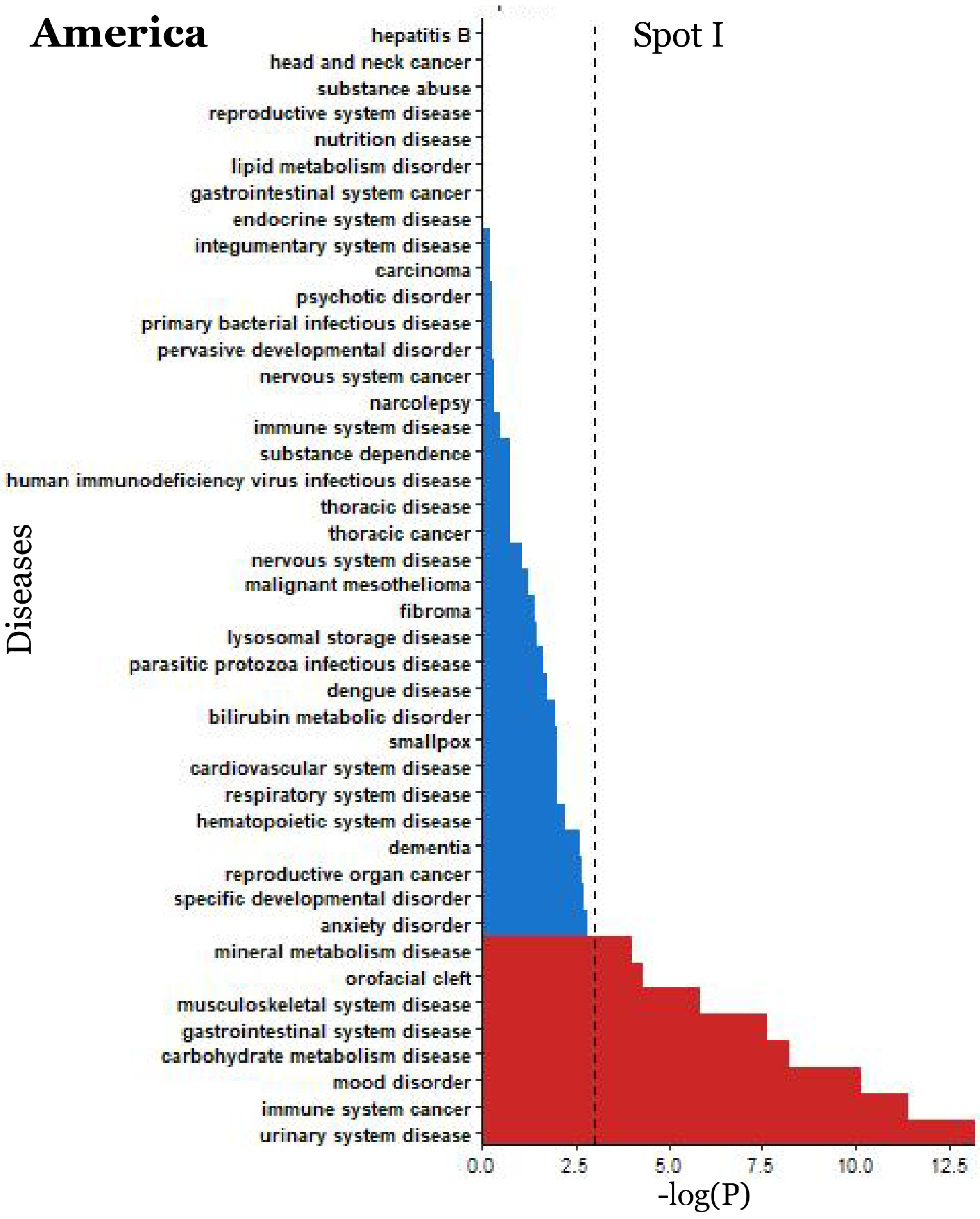

Supplementary Figure 15. Disease enrichment is the spots I.

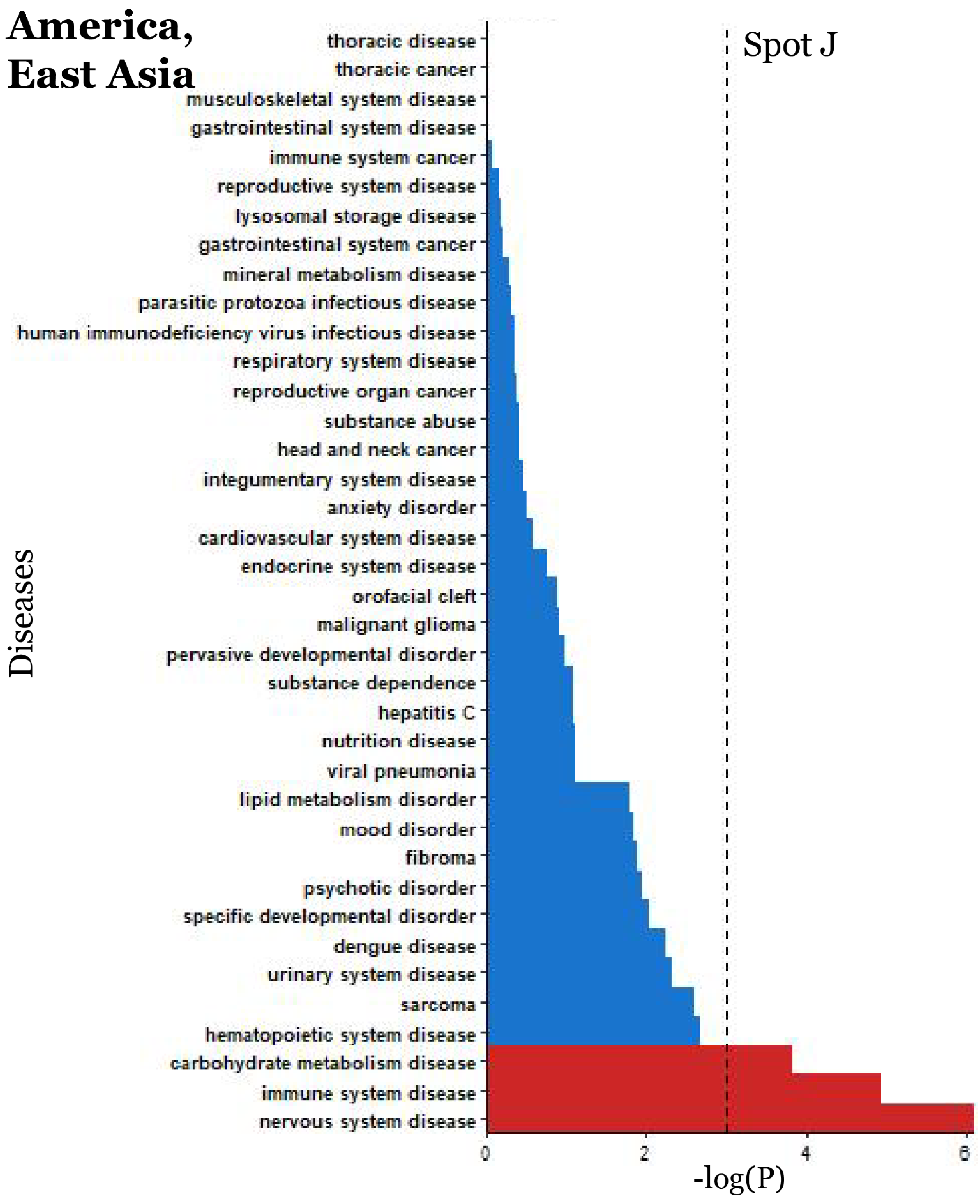

Supplementary Figure 16. Disease enrichment is the spots J.

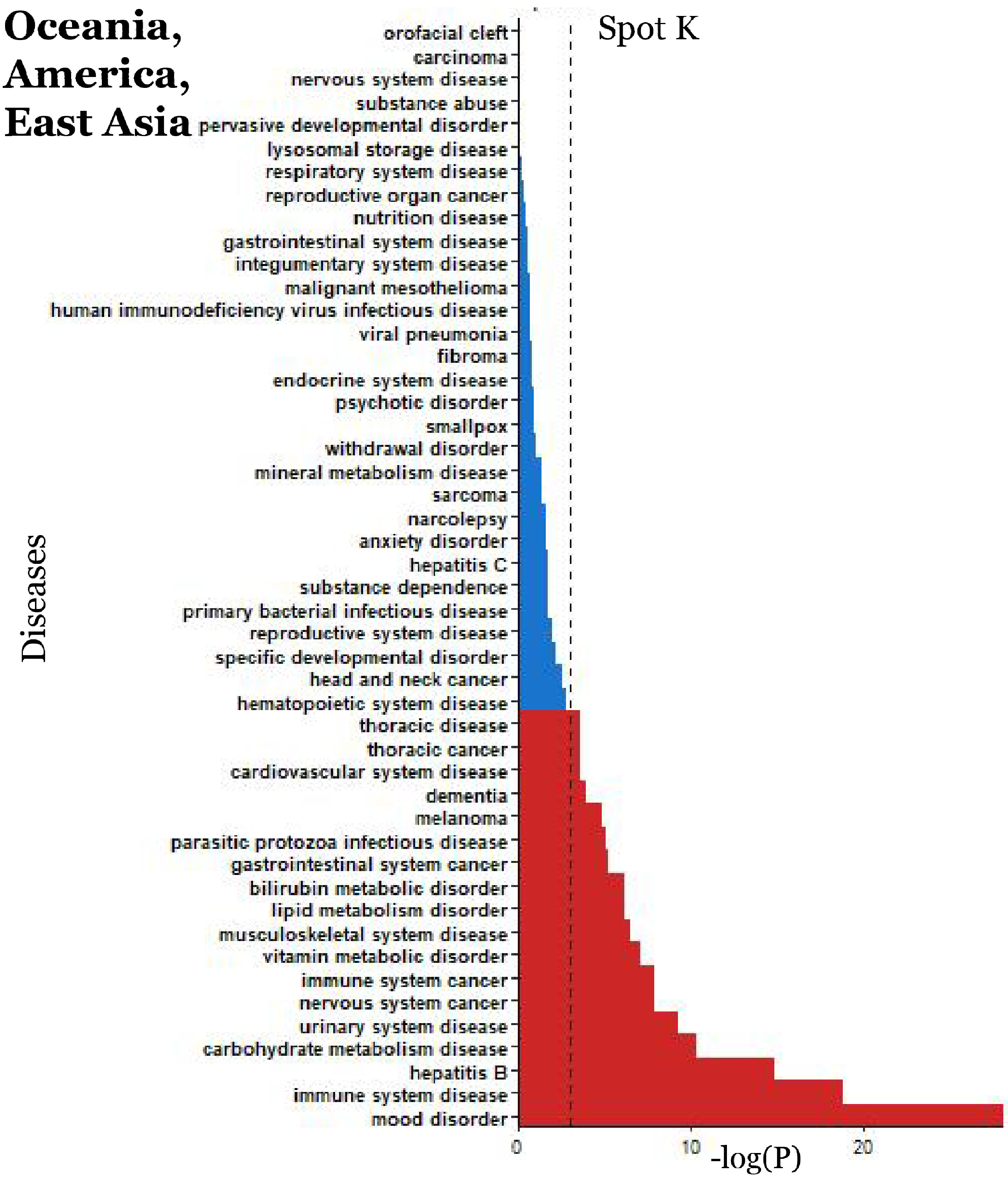

Supplementary Figure 17. Disease enrichment is the spots K.

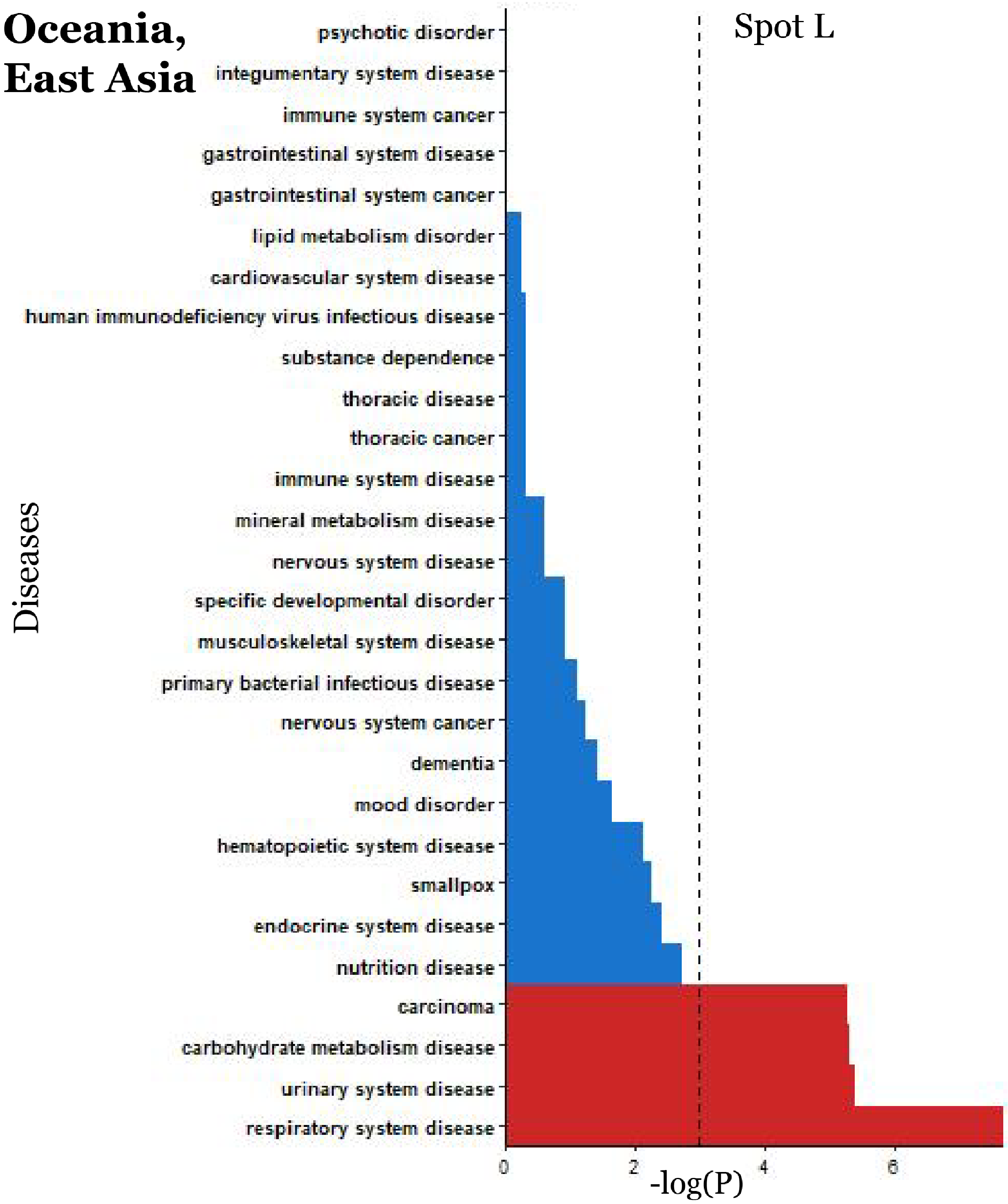

Supplementary Figure 18. Disease enrichment is the spots L.

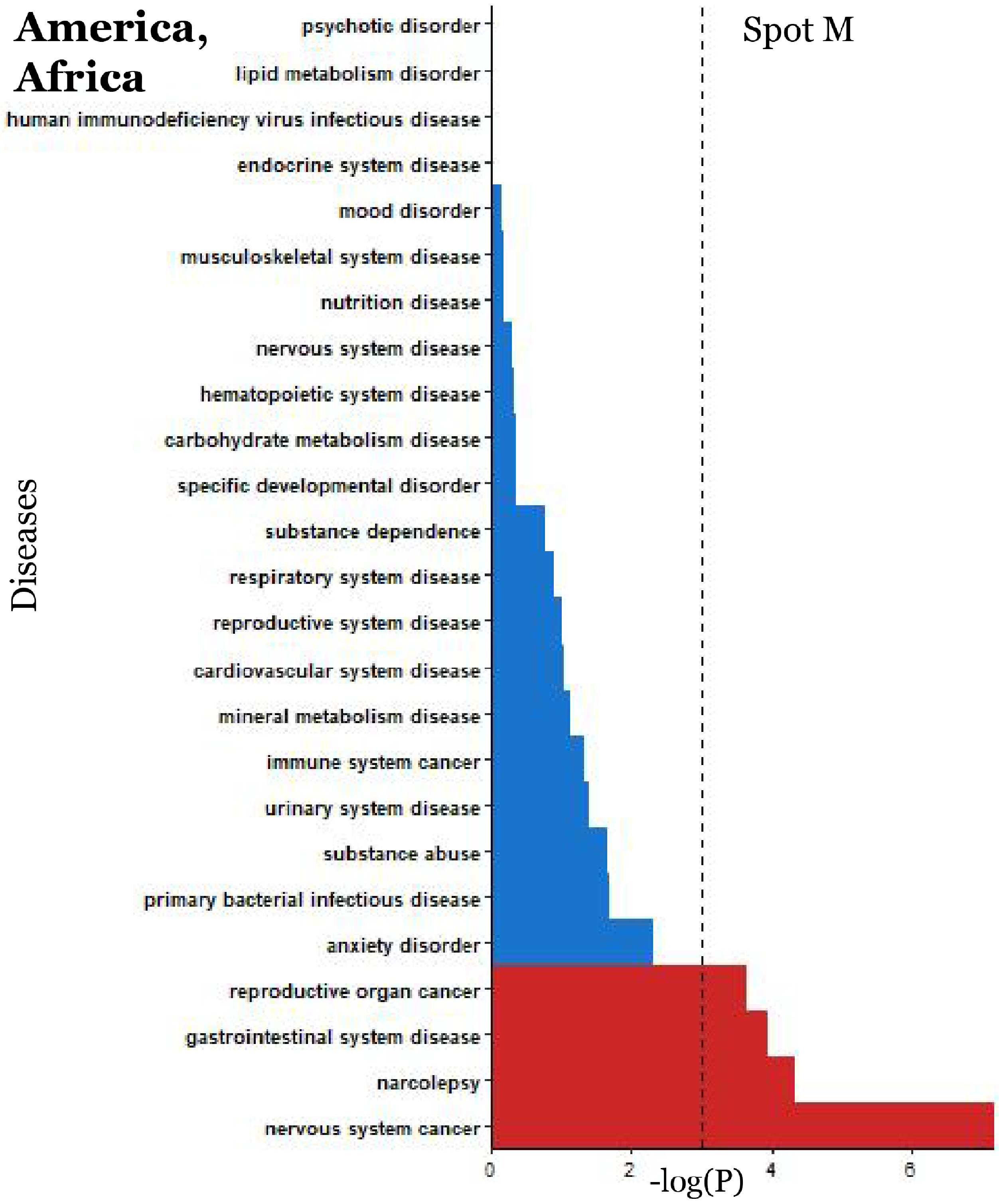

Supplementary Figure 19. Disease enrichment is the spots M.

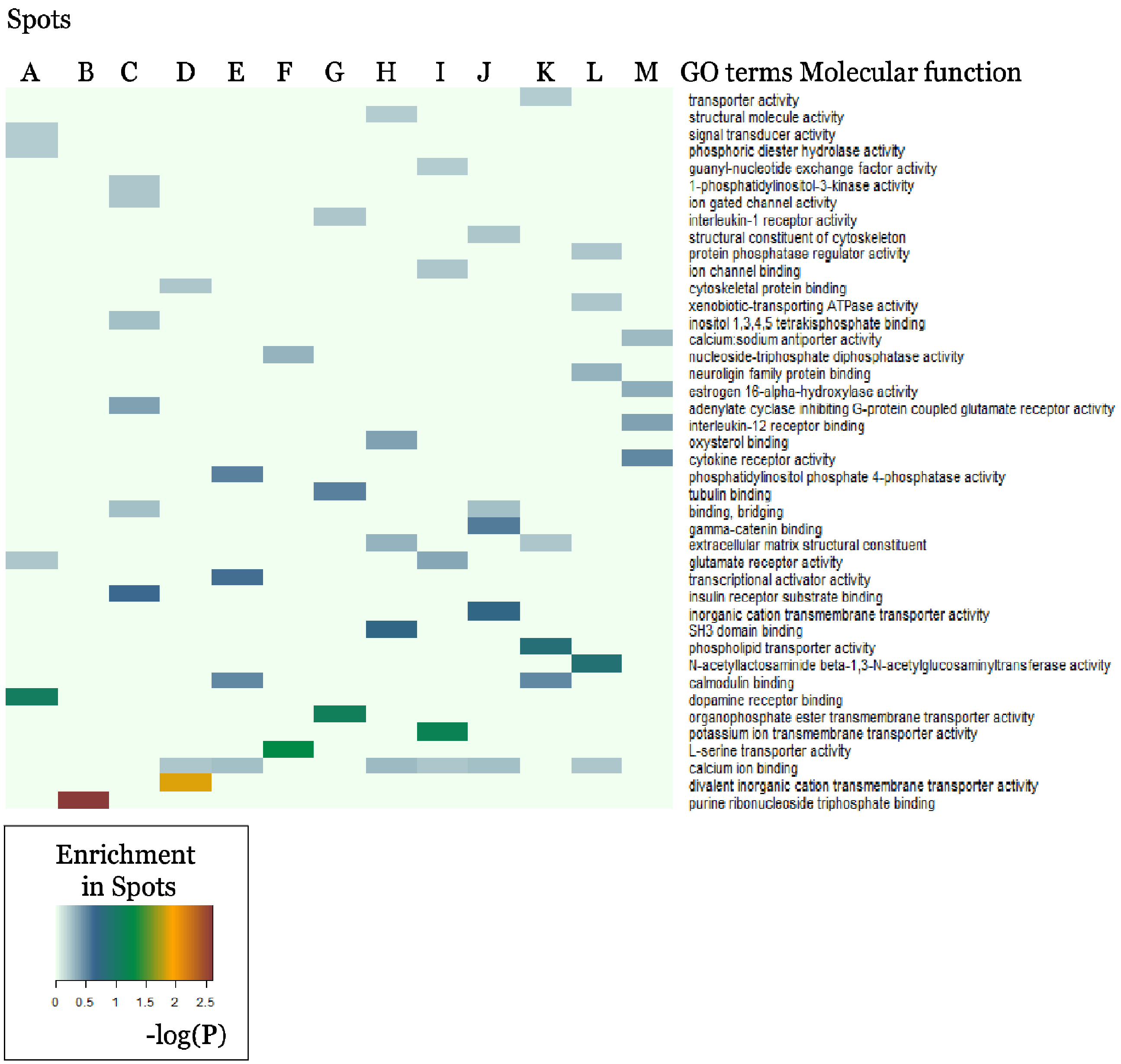

Supplementary Figure S20. Heatmap of enrichment of GO Molecular function terms in spots.
